# Melatonin regulates the periodic growth of secondary hair follicles through the nuclear receptor RORα

**DOI:** 10.3389/fvets.2023.1203302

**Published:** 2023-07-10

**Authors:** Zeyu Lu, Jing Wu, Jing Wu, Tiejia Zhang, Junyang Liu, Qing Mu, Terigele, Zixian Wu, Yanjun Zhang, Rui Su, Zhihong Liu, Zhiying Wang, Ruijun Wang, Lv Qi, Yanhong Zhao

**Affiliations:** ^1^State Key Laboratory of Animal Genetics and Breeding and Reproduction, College of Animal Science, Inner Mongolia Agricultural University, Hohhot, Inner Mongolia, China; ^2^College of Food Science and Engineering, Inner Mongolia Agricultural University, Hohhot, Inner Mongolia, China; ^3^Shangdu County Vocational and Technical School, Ulanqab, Inner Mongolia, China; ^4^Zhangbei Liang Mianjing People's Government, Zhangjiakou, Hebei, China

**Keywords:** melatonin, RORα, secondary hair follicle, hair follicles grow periodically, Inner Mongolia cashmere goat

## Abstract

Cashmere is the fine bottom hair produced by the secondary hair follicles of the skin. This hair is economically important. Previous studies by our research group have shown that exogenous melatonin (MT) can regulate the periodic growth of secondary hair follicles, induce the secondary development of villi, and alter the expression of some genes related to hair follicle development. Few studies on the regulation of villus growth by MT binding receptors have been published. In this study, MT was implanted subcutaneously behind the ear of Inner Mongolia cashmere goats. RT-qPCR, *in situ* hybridization, Western blot analysis, immunofluorescence and RNAi techniques were used to investigate the receptors and functions of MT in regulating the development of secondary hair follicles in Inner Mongolia cashmere goats. The results showed that MT binds to the nuclear receptor RORα on dermal papilla stimulates hair follicle development and promotes villus growth. The RORα mRNA expression in the skin of Inner Mongolia cashmere goats was periodic and showed a trend of first increasing and then decreasing. The expression began to increase in February, peaked in April, and reached the lowest level in May. RORα significantly affected the mRNA expression of β-*catenin* gene, a key gene in hair follicle development, in the presence of MT. It will lay a solid molecular foundation for further research on the regulation mechanism between MT receptor and villus growth and development and to achieve artificial regulation of villus growth time and yield to improve the effect of villus production.

## 1. Introduction

As an important textile material, cashmere is known as “fiber gem” and “soft gold” because of its light, soft and warm fiber and has economic value in animal husbandry. The skin of the Inner Mongolia cashmere goat is composed of cashmere and wool. The primary hair follicles develop into wool, and the secondary hair follicles develop into cashmere. The primary hair follicles generally appear at 55 days of gestational age and develop completely at approximately 135 days. Secondary hair follicles begin at 75 days of gestational age and are fully developed at 6 months after birth (1, 2). Hair follicle development is divided into three periods: the growth phase, catagen and telogen. The growth phase starts in April every year, and growth accelerates from August to September; during catagen, which starts in October, the hair stem stops growing, and epithelial cells begin to undergo apoptosis; during telogen, which occurs from December to March of the following year, hair follicle growth enters a relative resting phase (2).

The growth and development and periodic changes of hair follicles are complex processes regulated by many factors, including endocrine hormones and active molecules such as growth factors and cytokines, including melatonin (MT). MT is an indole hormone secreted mainly by the pineal gland and by some plants. Many studies have shown that MT has a wide range of functions, including immune regulation (3), treatment of animal diseases (4), antioxidant effects (5, 6), treatment of psychiatric diseases (7), use as a sleep aid (8, 9), resistance to radiation damage (10), antitumor effects (11), animal reproduction (12–15), induction of hair follicle development prior to the growth phase and other functions (16–19). In addition to directly acting on its receptors to alter circadian activity, MT can bind with highly chimeric sites in the pituitary to affect the secretion of nervous system hormones and then affect circadian activity. Ross et al. (20) found that the PER1 mRNA level in the pituitary of hamsters increased with the decrease in MT content in the blood.

Light intensity and sunshine duration had a major influence on MT secretion in animals, and MT content in the blood of animals changed periodically with the season. When the light duration was short, the content of MT in the blood increased and otherwise decreased. Studies have shown that MT can promote the villus growth of cashmere goats in the non-cashmere period. Since the nights are long and the days are short in winter, MT can promote the villus growth of cashmere goats and stimulate hair follicles to increase cashmere production (21, 22). MT was shown to have a regulatory effect on the periodic growth of villi of seasonal molting animals (23). Duan Chunhui (24) implanted MT in seasonal long-haired sheep in April and June and the results showed that MT could improve the wool yield and length of half-sibling sheep and non-half-sibling sheep and had a more obvious effect on promoting wool growth of half-sibling sheep. Wang Yan (25) found through qPCR that MT could activate Wnt signaling in catagen and telogen and improve the expression of Wnt10b. Through immunohistochemistry and HE staining, Mu Qing (26) found that after MT implantation, the number of secondary hair follicles in May and June was significantly higher than that in the control group, and the distance to the epidermis was closer than that in the control group, indicating that MT may activate related genes to restart and promote hair follicle development in advance and play an important role in the transition stage from telogen to the growth phase. Chang Zili (27) also showed that MT can induce the early occurrence of hair follicles, resulting in the secondary development of hair follicles, and MT downregulated the expression of Delta1, Notch1 and hairless in the Notch pathway during the growth phase, promoting villus growth.

Hormones function by binding to the corresponding receptor. MT is a highly lipophilic substance that easily crosses the cell membrane and nuclear membrane and can also be dissolved in aqueous solution; thus, it has various roles in tissue cells. Two classes of MT receptors have been identified thus far: membrane receptors (MT1, MT2, and possibly MT3) and nuclear receptors (RZR/ROR, a member of the orphan receptor subfamily). Mammalian MT receptors consist of MT1 (Mel1a), MT2 (Mel1b) and their related receptor GPR50 (28). Mel1c is the MT receptor of nonmammalian animals. Mel1a is mostly distributed in the hypothalamus, pituitary and suprachiasmatic nuclei (SCN), while Mel1b is mostly distributed in the retina (29, 30). MT secretion is increased at night, and MT binds to the G-protein-coupled receptor and activates MTNR1a, which enables the cells in the animal to detect radiant light energy and coordinate the circadian activities of cells within 24 h through the light reception of the retina, consistent with the natural photocycle (29). Mel1b has an antagonistic effect on MT and can resist the inhibitory effect due to the increase in MT concentration, which is opposite to the effect of Mel1a (30–36).

The MT nuclear receptor RORα, also known as the MT ligand, shares a set of DNA domains and ligand domains with RORβ and RORγ. A previous study showed that RORα1 could be activated by MT under specific conditions, and MT was identified as the ligand of RORα. Later, Wang et al. (37) found that 7A-hydroxyl and 24S-hydroxyl cholesterol could inhibit the transcriptional activity of RORα and RORγ in liver cells. LBD binding of RORγt small molecule inhibitors was shown to inhibit the function of Th17, which further confirmed that the natural ligands of RORα and RORγt are cholesterol and its derivatives (38). Studies have shown that CS mRNA binding to SR1001 in the liver can inhibit the circadian activity of RORα and CS mRNA (39) expression, suggesting that RORα plays a key role in circadian rhythm.

Breast (40), melanoma (41), hepatocellular carcinoma (42), and colon cancers (43), etc. have been reported to be closely related to RORα. Whether RORα is involved in MT regulation of villus growth has not yet been reported. In this study, real-time fluorescence quantitative PCR, *in situ* hybridization, RNAi and other methods were used to preliminarily identified RORα as the receptor of MT and the role of RORα in MT regulation of villus cycle growth.

## 2. Materials and methods

### 2.1. Animals

**Figure 1:**
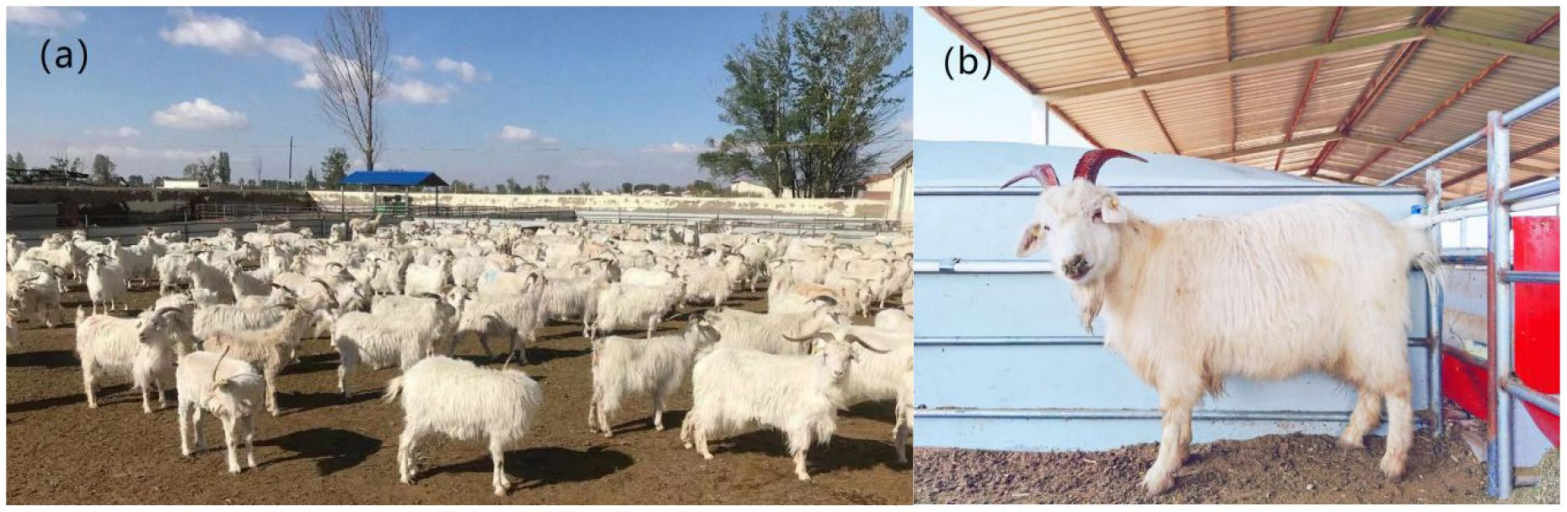
Images of 1-year-old Inner Mongolian cashmere goats. Photo taken of Inner Mongolia cashmere goats from Inner Mongolia Jinlai Animal Husbandry Technology Co., Ltd. **(A)** Inner Mongolia cashmere goats, **(B)** Inner Mongolia cashmere goat.

One-year-old Inner Mongolia Albasi cashmere goats were obtained from Jinlai Animal Husbandry Technology Co., Ltd., Inner Mongolia Autonomous Region and were fed a mixture of coarse feed and granular concentrate according to the sheep farm feeding standard (400 g/d, coarse feed 50%, granular concentrate 50%). The animal management and operation procedures adopted in this study were in accordance with the guidelines of the Animal Feeding and Use Committee of Inner Mongolia Agricultural University and were carried out in strict accordance with animal welfare and ethical guidelines. The animals are shown in Figure 1.

### 2.2. Implantation of MT

The growth cycle of cashmere was 12 months. MT capsules were implanted in the subcutaneous tissue behind the ears of the cashmere goats every 2 months (provided by the Animal Science Laboratory of Northeast Forestry University), and the amount implanted was 2 mg/kg for each sheep.

### 2.3. Sample collection

All methods were implemented in accordance with the relevant guidelines and regulations of the European Commission (1997). The method was also approved by the Ethics Committee of Inner Mongolia Agricultural University. The animals used in this test were from Inner Mongolia Jinlai Animal Husbandry Technology Co., Ltd. Disinfect and sterilize the sampling environment and sampling tools, and the sampling personnel wear experimental clothing, masks, gloves, etc. to prevent sample contamination. One-year-old female Inner Mongolia cashmere goats with similar body weights were randomly divided into two groups: the experimental group (MT) and the control group (no preparation). Local anesthesia with procaine was used to minimize pain. Skin tissue was collected from the experimental group and the control group from the posterior edge of the shoulder every month for a year. The goats with skin injury were observed for 1 week to confirm their recovery. Each skin sample was approximately 1 cm^2^, part of which was transported back to the laboratory in liquid nitrogen to extract total RNA (to synthesize cDNA) and total protein from the sample. The other part was placed in 4% paraformaldehyde and transported to the laboratory for preparation of paraffin sections. A cashmere goat was randomly selected and slaughtered 4 h after sunset on the day of skin tissue collection. The head was collected, the midline skin was cut lengthwise along the nasal bone and peeled off laterally until the skull was completely exposed, and a cross cut was made in the middle of the nasal bone until the brain tissue inside the head was observed. To prevent damage to the cerebral cortex, we cut the bone on either side through a cross incision, flipped the skull cap 180° to expose brain tissue, and moved the cerebellum aside to expose the junction between brain stem and brain, allowing visualization of the hypothalamus of the cashmere goat. The hypothalamus was clipped along the root, and the hypothalamus tissue was rapidly transported back to the laboratory in liquid nitrogen to extract total RNA (for synthesis of cDNA) from the sample. Mouse skin tissue cDNA was provided by the State Key Laboratory of Animal Genetics, Breeding and Reproduction.

### 2.4. Primer design

Based on the MTNR1a, MTNR1b, RORα, *SFRP1, TCHHL1*, and *β*-*catenin* DNA sequences of other species and goat sequences in GenBank (Nut Cloud link^1^), the conserved cDNA sequences of other species were obtained by DNAStar homology comparison. Primers were designed according to the transitron principle, and *β*-actin was used to correct the expression level (Table 1).

**Table 1:** Primer sequences.

Gene name	Forward primer	Reverse primer	Target fragment (bp)
MTNR1a	5’-CTGTCCGTGTATCGGAACAAG-3’	5’-CCTGGGGCTTTAGTTTCGGTTTGT-3’	550
MTNR1b	5’-GCAACCTCCTGGTCATCCT-3’	5’-GACCACGCTCAGACCCAT-3’	238
RORα	5’-GCCGCTGACTCCCACCTAT-3’	5’-ATCACCTCCCGCTGCTTG-3’	417
*β-actin*	5’-GTCACCAACTGGGACGACA-3’	5’-AGGCGTACAGGGACAGCA-3’	208
*SFRP1*	5’-GCACGACCGTGTGTCCTCCATGT-3’	5’-GCTTCTTCAGCTCCTTCTTCTTGAT-3’	192
*TCHHL1*	5’-GCCAGAAAGTGGCCCAAGATGTAT-3’	5’-CTCCAAACCATTCTCCTGTCTCAGT-3’	100
*β-catenin*	5’-GACCACAAGCAGAGTGCT-3’	5’-TGTCAGGTGAAGTCCTAAA-3’	192

### 2.5. *In situ* hybridization

*In situ* hybridization was performed on skin tissue sections to detect the position of complementary sequences in eukaryotic cells using RNA probes of known sequences. Based on the 550 bp MTNR1a cDNA, a 364 bp MTNR1a probe PCR primer was designed. The RORα probe template was obtained by PCR amplification and purification using the RORα plasmid as a template. The localization of MTNR1a and RORα in Inner Mongolia cashmere goat skin was studied by specifically combining a DIG-labeled nucleic acid probe with cashmere goat skin tissue according to the principle of base complementation.

### 2.6. Immunofluorescence staining detection of RORα protein expression in hair follicles

The skin tissue was cut into 0.15 cm^3^ pieces and placed in PBS containing 30% sucrose for dehydration. After the slicer was precooled, the tissue pieces were cut into 8 μm thick frozen sections and sealed with PBS buffer of 10% BSA (containing 0.4% Triton to increase permeability) at room temperature and dark conditions for 1 h. The slices were incubated with 0.02 mol/L primary antibody (Anti-ROR alpha, ab60134, Abcam, America) containing 0.4% Triton prepared with PBS at 4°C overnight and then incubated with 0.01 mol/L secondary antibody [Goat Anti-Rabbit IgG H&L (Alexa Fluor®) preabsorbed, ab150081, Abcam, America] containing 0.4% Triton prepared with PBS at room temperature for 1 h. Rinse with 0.01 mol/L PBS after each step. The sections were dried naturally and sealed for fluorescence microscopy to detect the location of RORα protein in skin hair follicles.

### 2.7. Western blot

Total protein was extracted and isolated from the skin tissues of goats with and without MT implantation in February, September and December and electrically imprinted onto a PVDF (polyvinylidene fluoride) membrane. After being sealed at room temperature for 1 h, the membrane was incubated with primary antibody overnight. After incubation, the membrane was washed with 15 ml of TBS/T. After incubation with secondary antibody for 1.5 h, the membrane was rinsed again, and the protein expression of RORα in skin hair follicles was detected.

### 2.8. Cell culture

Fibroblasts were cultured by the tissue block method. The sample was washed 3–5 times with PBS containing 3% double antibody. Tissue blocks were cut into 2 mm^2^, placed in a 25 mm^2^ culture flask, flipped until tissue block faced up, and placed in an incubator at 37°C and 5% CO2 for 4 h. After removal, the samples were turned over, complete medium was added to soak the tissue blocks, and the samples were returned to the incubator and continued to grow. The medium was changed every 3 days according to the growth state of the cells. Fibroblasts were cultured as donor cells, and the cells were inoculated in 24-well plates at a density of 5 × 10^3^ cells/ml. The cells were counted with a blood cell counter for 8 consecutive days. The cell number in 3 wells at each time point was calculated, and the average value was used to generate the cell growth curve.

### 2.9. The effect of RNAi on the expression of RORα and other genes in fibroblasts

A RORα-specific shRNA vector was constructed, and the RORα target sequence was synthesized according to RORα sequence and interference fragment design principles (Table 2). An shRNA template was used for the annealing reaction, restriction enzyme digestion of the vector and target fragment was performed, the reaction product and linearized plasmid were connected and transformed into receptive cells, and positive clones were identified and resequenced. GFP-labeled RORα shRNA cells were transfected, and RORα mRNA expression was detected by RT-qPCR.

**Table 2:** RORα target sequence.

shRNA	Target sequence	
shRNA1	5’-CACCGTCTTGATATCAATGGAATCATTCAAGAGATGATTCCATTGATATCAAGACTTTTTTG-3’	5’-AGCTCAAAAAAGTCTTGATATCAATGGAATCATCTCTTGAATGATTCCATTGATATCAAGAC-3’
shRNA2	5’-CACCGAAACTTGCCAATACTTGAGATTCAAGAGATCTCAAGTATTGGCAAGTTTCTTTTTTG-3’	5’-AGCTCAAAAAAGAAACTTGCCAATACTTGAGATCTCTTGAATCTCAAGTATTGGCAAGTTTC-3’
shRNA3	5’-CACCGTGGTATTTATCAGAATGTGCTTCAAGAGAGCACATTCTGATAAATACCACTTTTTTG-3’	5’-AGCTCAAAAAAGTGGTATTTATCAGAATGTGCTCTCTTGAAGCACATTCTGATAAATACCAC-3’
NC	5’-CACCGTTCTCCGAACGTGTCACGTCAAGAGATTACGTGACACGTTCGGAGAATTTTTTG-3’	5’-AGCTCAAAAAATTCTCCGAACGTGTCACGTAATCTCTTGACGTGACACGTTCGGAGAAC-3’

### 2.10. Statistical analysis

CT values of the number of cycles in each group were determined by comparative CT value method in relative quantification method. mRNA expression levels were analyzed and compared by 2^**-(ΔΔCT)**^ quantitative analysis method, and expressed in the form of mean ± standard error. SAS software was used for one-way ANOVA and significance analysis, *P* < 0.05 was considered statistically significant.

## 3. Results

### 3.1. Expression of MTNR1a, MTNR1b, and RORα in different tissues of cashmere goats

**Figure 2:**
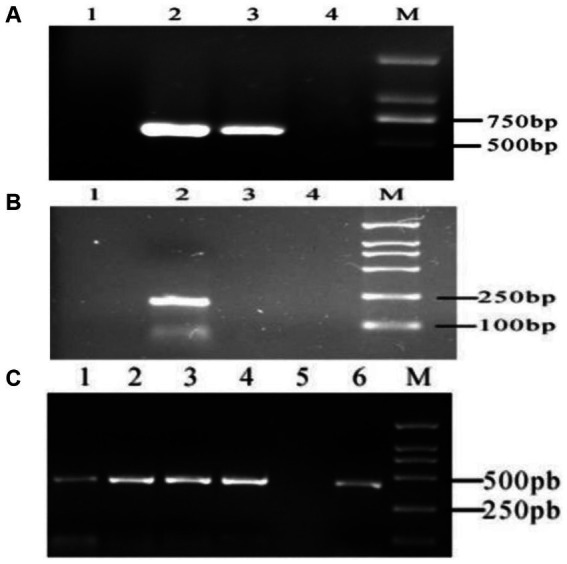
RT-PCR electrophoretic map. **(A)** MTNR1a M: DL2000 DNA marker. 1: Skin amplification results. 2, 3: Results of hypothalamus amplification. 4: Negative control. **(B)** MTNR1b M: DL2000 DNA marker. 1: Negative control. 2: Results of mouse skin amplification. 3: Results of hypothalamus amplification in cashmere goats. 4: Results of skin amplification in cashmere goats. **(C)** RORα M: DL2000 DNA marker. 1–4: Results of skin amplification in cashmere goats. 5: Negative control. 6: Results of amplification of the hypothalamus from cashmere goats.

According to the above primer design principle, the specific MTNR1a cDNA band from the cashmere goat hypothalamus was amplified. No target fragment of MTNR1a cDNA was amplified from cashmere goat skin (Figure 2A). The MTNR1b fragment could not be amplified from either goat skin or hypothalamus cDNA, but specific bands were amplified from mouse skin cDNA (Figure 2B). The RORα fragment was amplified from the cDNA of both tissues (Figure 2C). From the above results, we preliminarily concluded that MTNR1a is expressed in the hypothalamus of cashmere goats but not the skin. MTNR1b was not expressed in the skin and hypothalamus of cashmere goats. RORα was expressed in the skin and hypothalamus of cashmere goats in each month.

### 3.2. *In situ* hybridization was used to detect the expression of MTNR1a in different tissues of cashmere goats

**Figure 3:**
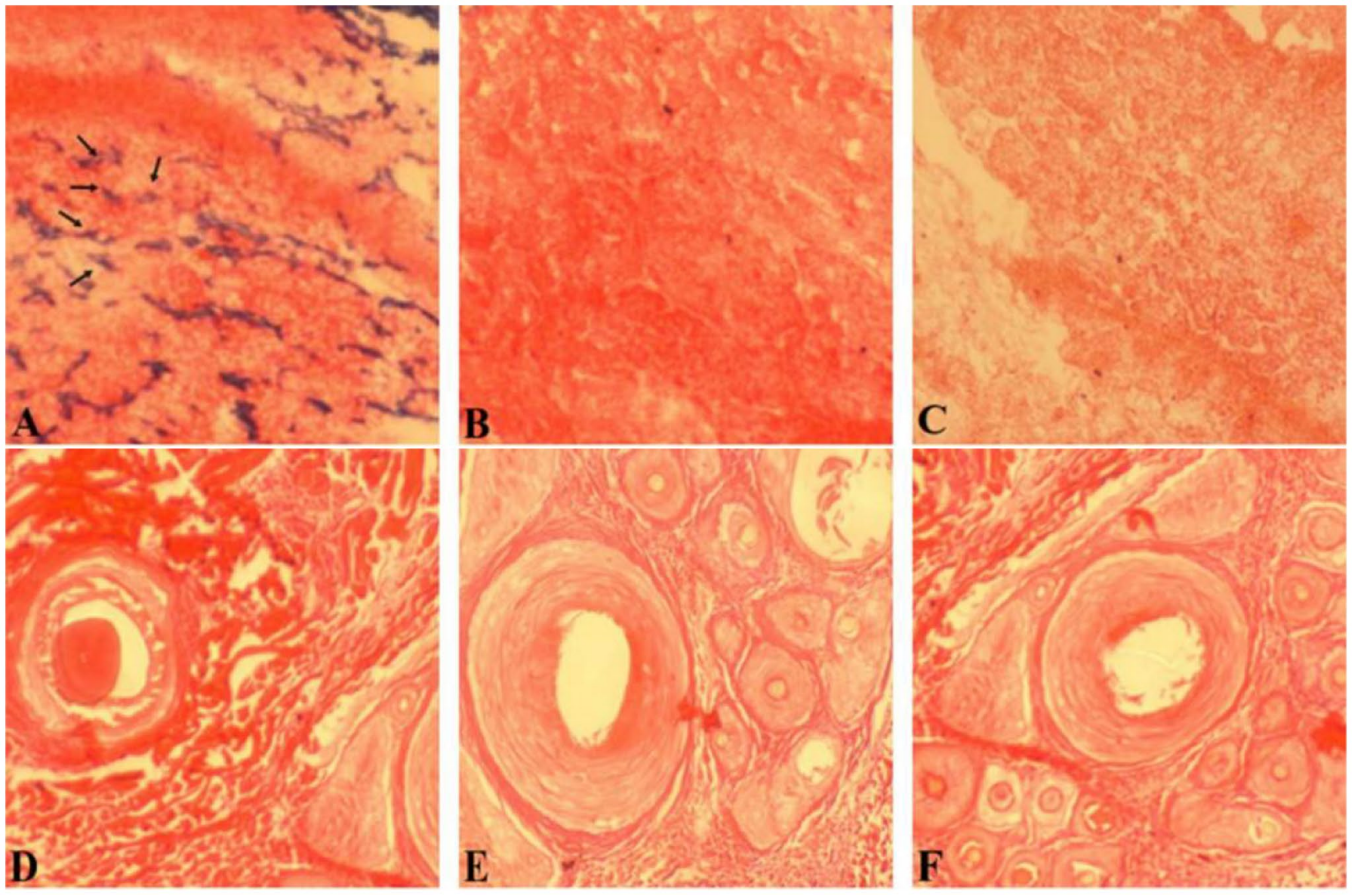
Results of *in situ* hybridization of the MTNR1a cDNA probe in different tissues. **(A)** An antisense strand probe was hybridized *in situ* in hypothalamus tissue. **(B)** The sense strand probe was hybridized *in situ* in hypothalamus tissue. **(D)** The antisense strand chain probe was hybridized *in situ* in skin tissue. **(E)** The sense strand probe was hybridized *in situ* in skin tissue. **(C,F)** Blank control. Magnification is 100×.

The amplified sequences of the hypothalamus and skin were transcribed into MTNR1a cDNA probes, and FISH detection was performed. The results showed a strong positive signal in the hypothalamus, but no positive signal was detected in the skin (Figure 3), indicating that MTNR1a was widely expressed in the hypothalamus tissue but not in the skin tissue.

### 3.3. RORα mRNA expression changed dynamically during the villus development cycle

**Figure 4:**
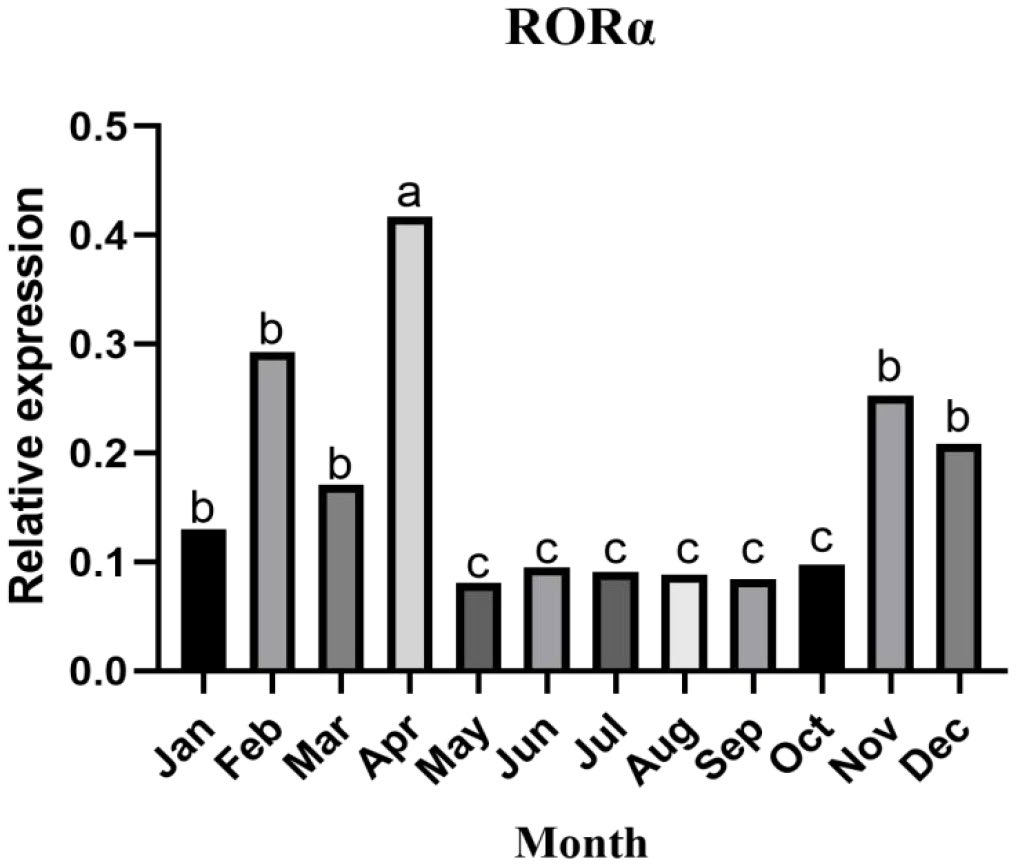
The relative expression of RORα mRNA in the skin of cashmere goats in different months. The expression of RORα mRNA changed dynamically throughout the villus development cycle. qPCR results are the control (*n* = 3). a, b, and c represent significant differences (*p* < 0.05).

In skin samples from Inner Mongolia cashmere goats, the target fragments of RORα and β-actin were 417 and 208 bp, respectively, and no bands were found in the negative control. The changes in RORα mRNA expression in different months showed that RORα mRNA expression changed dynamically during the villus development cycle (Figure 4), with the highest expression in April, followed by February, and the lowest expression in May. In general, the expression level was low in the growing stage and increased in catagen and telogen, with a significant difference.

### 3.4. *In situ* hybridization was used to detect RORα expression in different parts of cashmere goat skin

**Figure 5:**
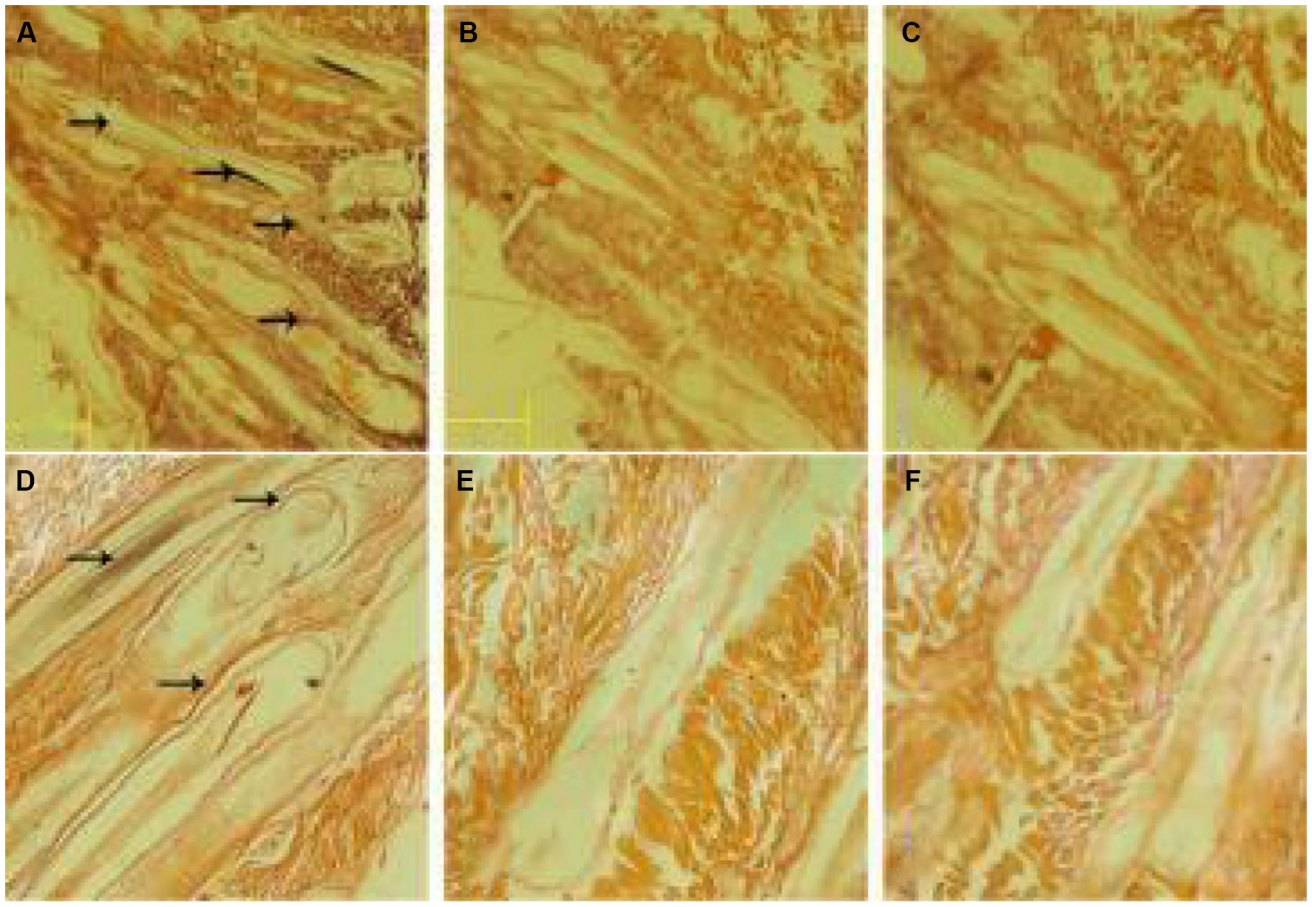
*In situ* hybridization of the RORα mRNA probe in skin tissue. **(A)** Results of *in situ* hybridization of the antisense strand probe in skin tissue of the MT-implanted group in February. **(B)** Results of *in situ* hybridization of the sense strand probe in skin tissue of the MT-implanted group in February. **(D)** Results of *in situ* hybridization of the antisense strand probe in skin tissue of the MT-implanted group in August. **(E)** Results of *in situ* hybridization of the sense strand probe in skin tissue of the MT-implanted group in August. **(C,F)** Blank control. Magnification is 40×.

*In situ* hybridization of hair follicles from cashmere goat skin in February, April, August, October, and December and hair follicles from cashmere goat skin implanted with MT was carried out by using a RORα cDNA probe. The distribution of RORα in skin hair follicles in February during the regression period, August during the growth period and the implantation group was randomly selected (Figure 5). Strong positive expression signals were found in the two different months and the implanted group. In February, strong positive signals were detected in the hair stem. In August, strong expression was observed in the hair stem cortex and outer root sheath. In the MT group, the positive signals of hair follicles were mainly found in the outer root sheath of the hair shaft and hair shaft.

### 3.5. Expression distribution of RORα in skin hair follicles

**Figure 6:**
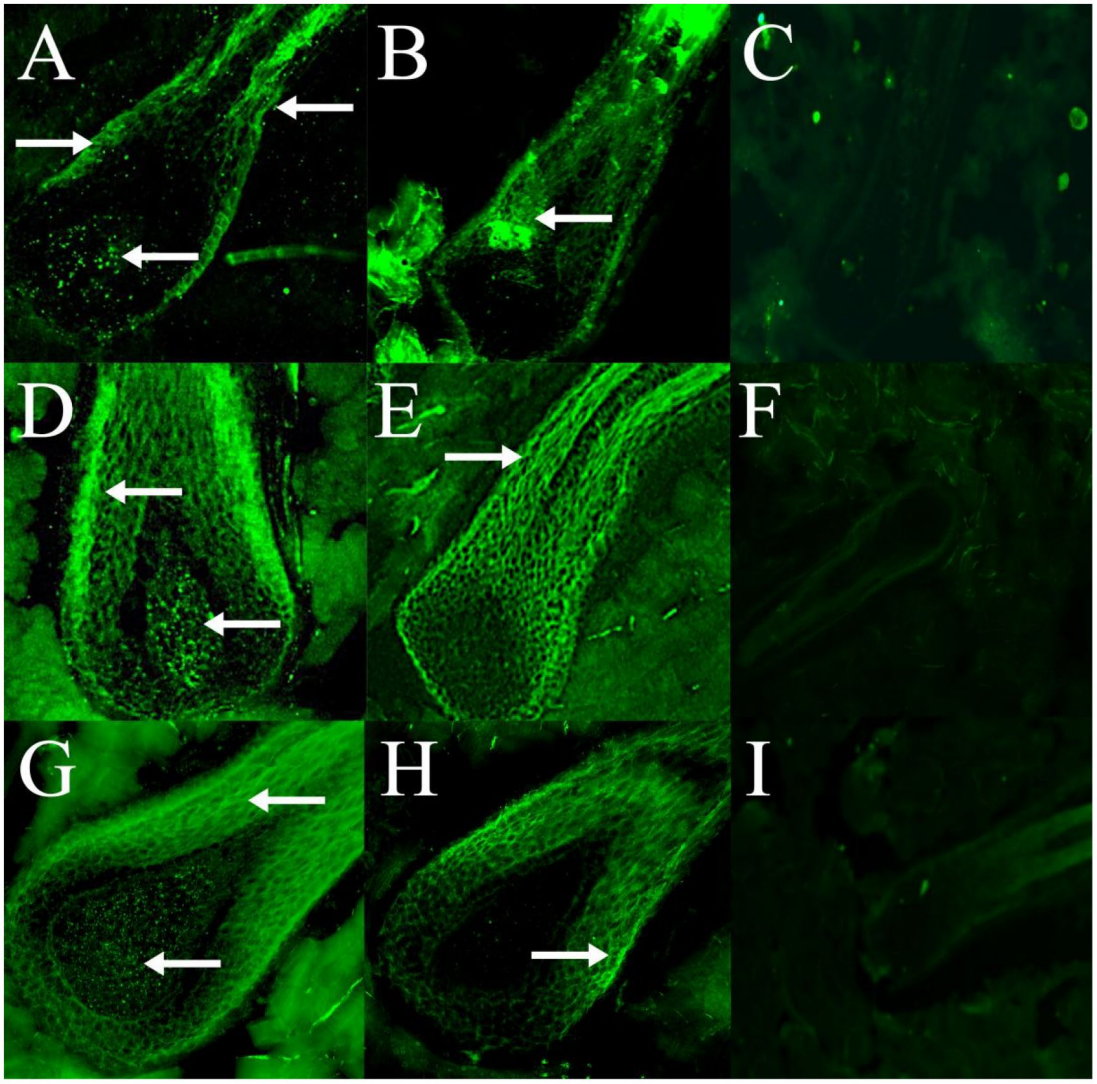
The expression and distribution of RORα protein in primary hair follicles in February, September and December were detected by immunofluorescence. **(A–C)** The expression of RORα protein in primary hair follicles of the experimental group, control group and negative control group in February. **(D–F)** The expression of RORα protein in primary hair follicles of the experimental group, control group and negative control group in September. **(G–I)** The expression of RORα protein in primary hair follicles of the experimental group, control group and negative control group in December. The magnification of **(A–E,G,H)** is 400×, **(F)** is 200×, **(I)** is 100×. The white arrow points to a fluorescent signal.

**Figure 7:**
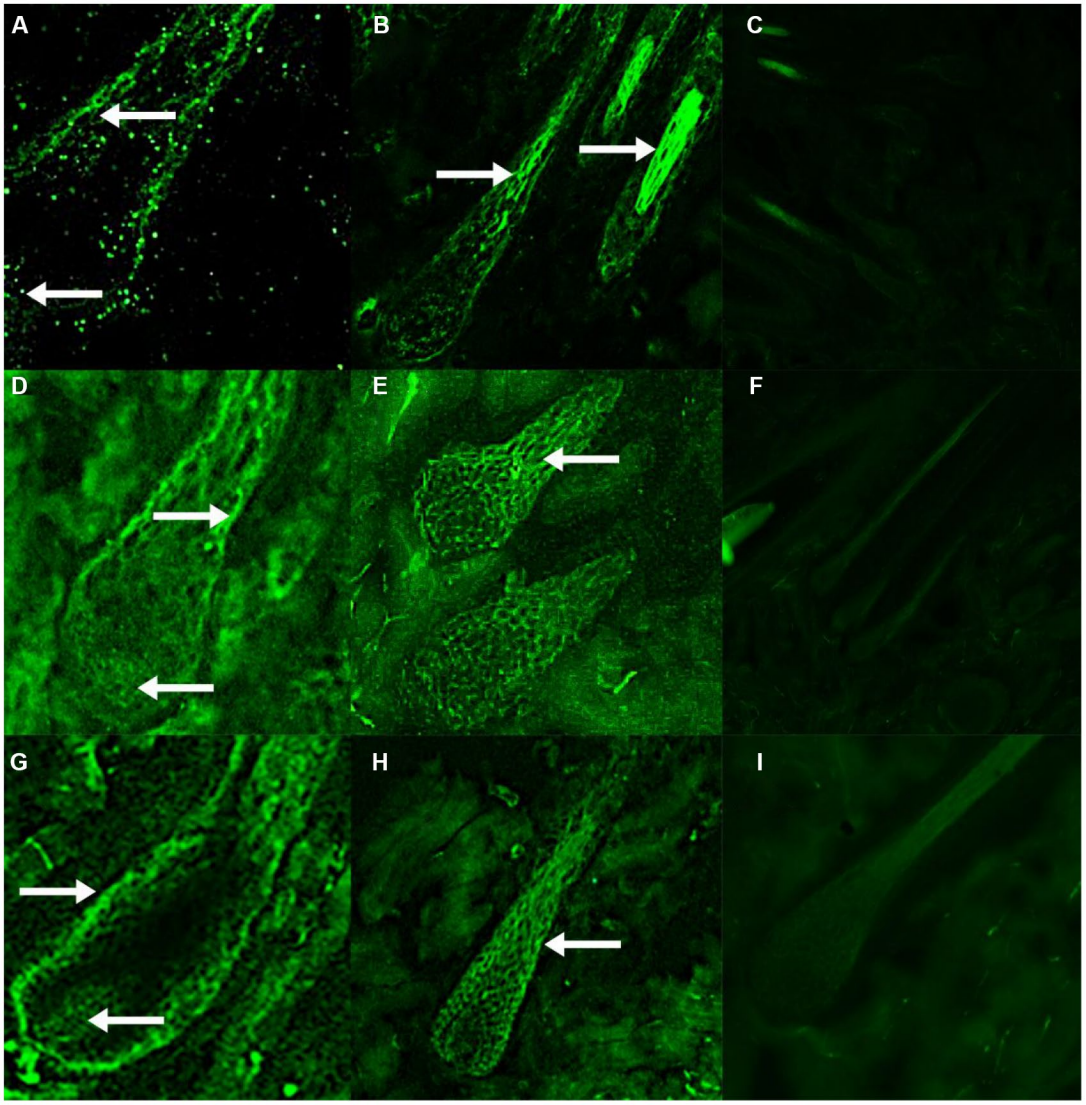
The expression and distribution of RORα protein in secondary hair follicles in February, September and December were detected by immunofluorescence. **(A–C)** The expression of RORα protein in secondary hair follicles of the experimental group, control group and negative control group in February. **(D–F)** The expression of RORα protein in secondary hair follicles of the experimental group, control group and negative control group in September. **(G–I)** The expression of RORα protein in secondary hair follicles of the experimental group, control group and negative control group in December. The magnification of **(A,D,G)** is 400×, **(E,H)** is 200×, **(B,C,F,I)** is 100×. The white arrow points to a fluorescent signal.

Immunofluorescence results showed that RORα was expressed in February, September and December of the hair follicle development cycle, and in February, September, and December RORα was expressed in telogen, the growth stage and catagen, respectively. In the MT-implanted group, RORα protein of primary hair follicles and secondary hair follicles was expressed in the external root sheath and dermal papilla of the hair follicle at three stages, while RORα protein of primary and secondary hair follicles in the control group was only expressed in the external root sheath of the hair follicle at three stages (Figures 6, 7). We concluded that the binding of MT and RORα on dermal papilla stimulates the development of hair follicles and promotes villus growth.

### 3.6. MT upregulated RORα protein expression in skin hair follicles

**Figure 8:**
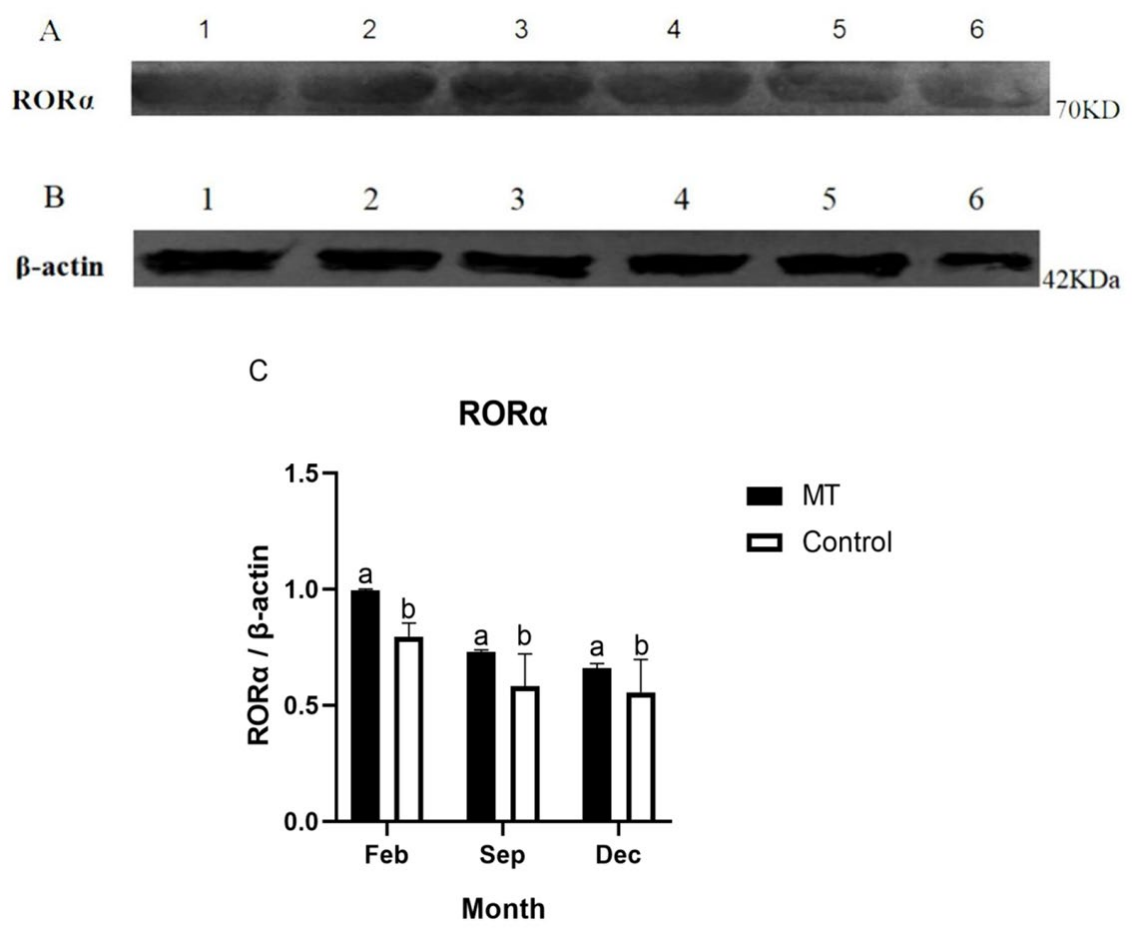
The protein expression of RORα and β-actin in the hair follicle development period of Inner Mongolia cashmere goats was detected by Western blotting. **(A)** 1, 2, and 3 are RORα proteins in the implanted MT groups of February, September, and December, respectively; 4, 5, and 6 are RORα proteins of the control group in February, September, and December, respectively. **(B)** 1, 2, and 3 are β-actin proteins in the implanted MT groups of February, September, and December, respectively; 4, 5, and 6 are β-actin proteins of the control group in February, September, and December, respectively. **(C)** Relative protein expression level of RORα in hair follicles in February, September, and December. The data are presented as the means ± SEMs of triplicates from three independent experiments, using fold change compared to the control group (*n* = 3). The measure of *β*-actin was used as Intrinsic reference protein for Western blot. a, b represent significant differences (*p* < 0.05).

To further verify the effect of MT on RORα protein, we used Western blotting to detect the changes in RORα protein in February, September, and December (Figures 8A,B). The expression of RORα in the implanted group was significantly higher than that in the control group in February and showed a downward trend from September to December, with no significant difference compared with the control group (Figure 8C). This result indicated that MT can upregulate RORα expression in February.

### 3.7. RNAi inhibited RORα expression in fibroblasts

**Figure 9:**
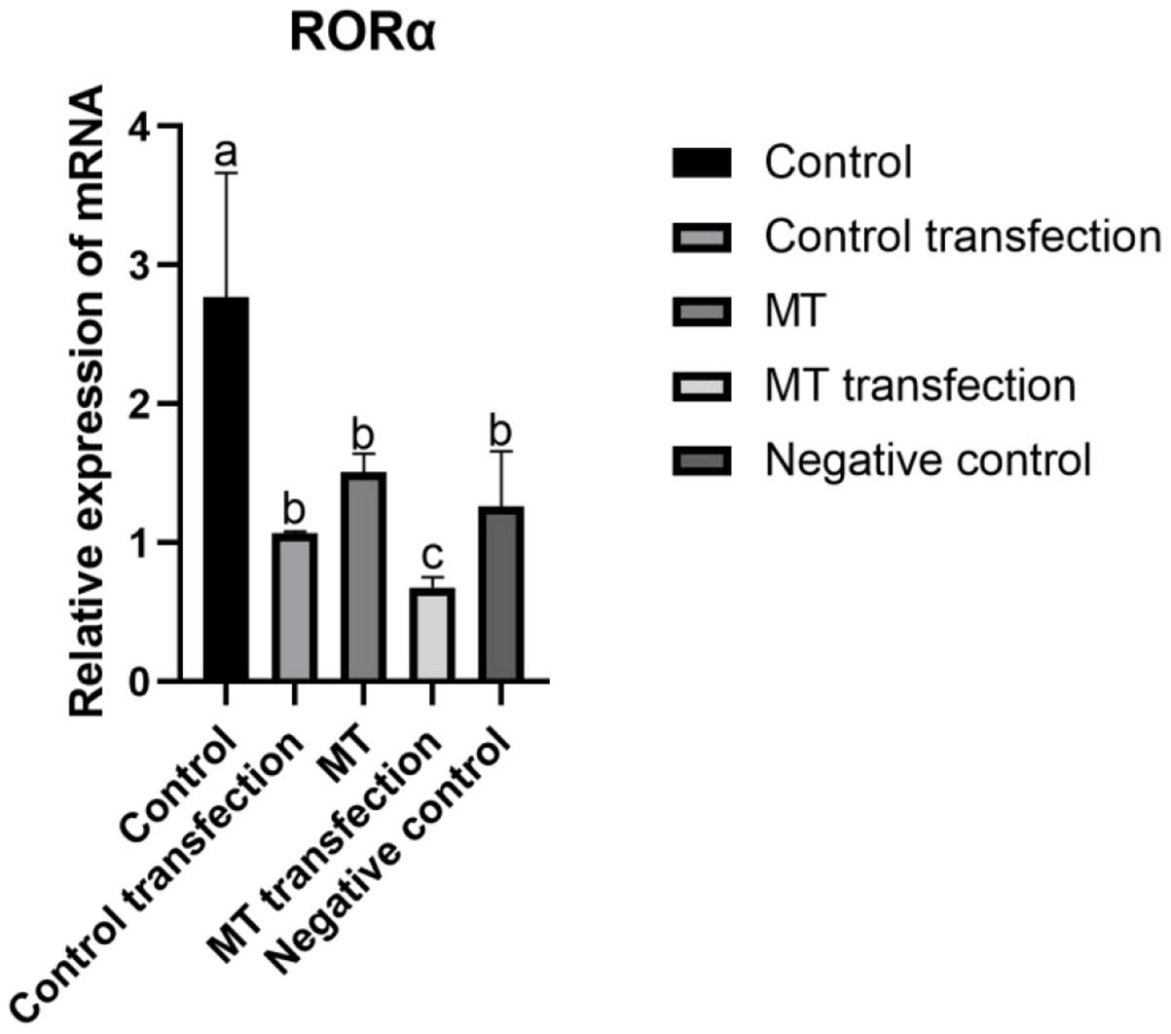
The effect of RNA interference on RORα gene expression in Inner Mongolia cashmere goat skin fibroblasts was detected by qPCR. shRNA was loaded into Inner Mongolia cashmere goat skin fibroblasts by RNA interference technology, and the expression of RORα was detected by qPCR before and after transfection in different groups. qPCR results are presented as means ± SEMs of triplicates from three independent experiments, using fold change compared to the control (*n* = 3). a, b, c represent significant differences (*p* < 0.05).

shRNA targeting the RORα gene was successfully constructed by a chemical synthesis method. After transfection with liposomes into fibroblasts, the transfection efficiency of the RORα shRNA was detected by RT-qPCR. The relative RORα mRNA expression was determined by the 2^**-(ΔΔCT)**^ method. However, the expression level of RORα in the transfected control group and the embedded fibroblasts was much lower than before transfection, and there was no significant difference between the negative control group and the implanted group (Figure 9). The results showed that MT significantly inhibited RORα.

### 3.8. The effect of RORα on *SFRP1, TCHHL1*, and *β*-*catenin* expression

**Figure 10:**
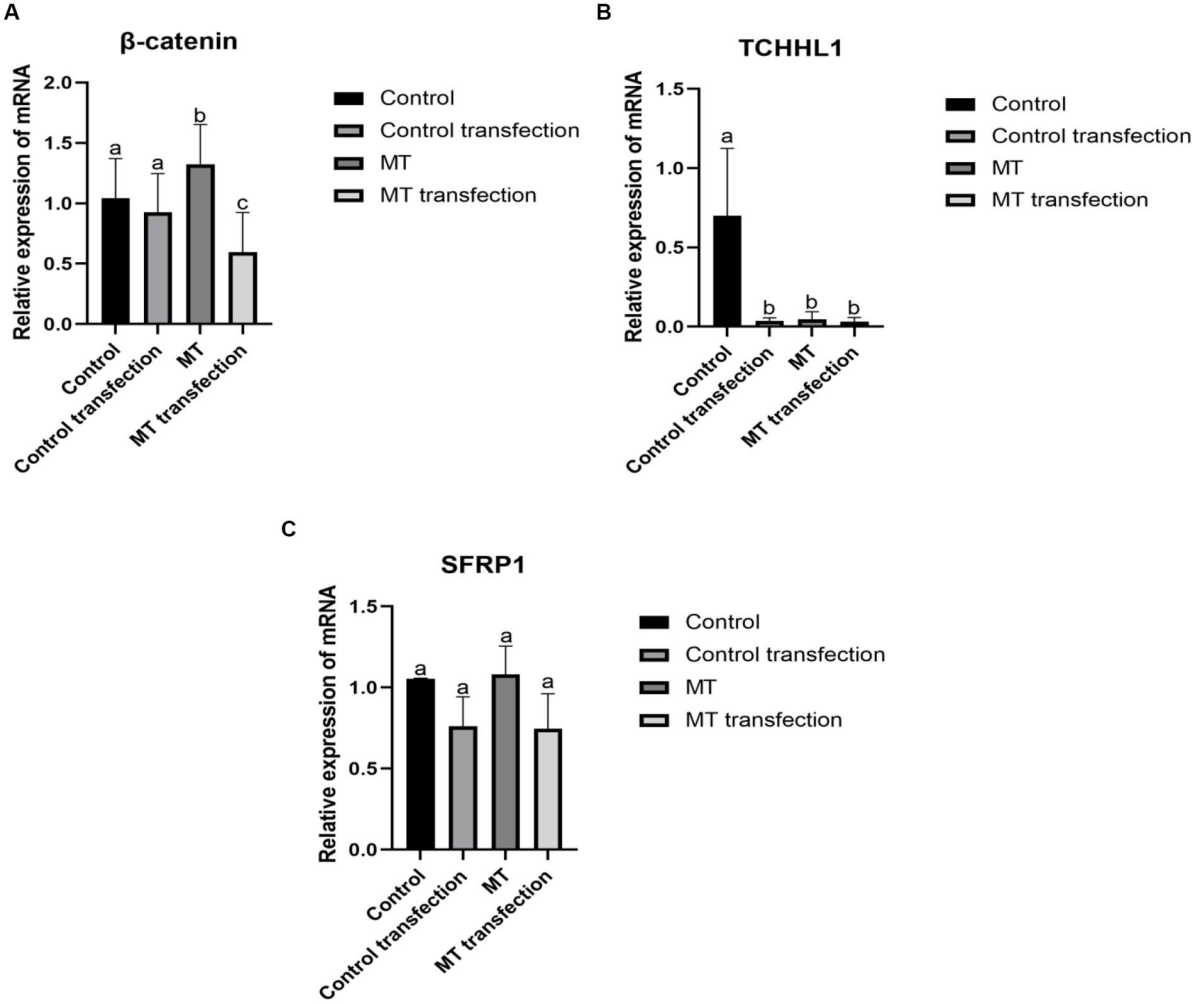
Effect of downregulation RORα expression on its related genes. **(A)** RNA interference inhibited RORα expression, and qPCR showed that MT could increase the expression of β-*catenin*. **(B)** RNA interference inhibited RORα expression, and qPCR showed that MT could lower the expression of TCHHL1. **(C)** RNA interference inhibited RORα expression, and qPCR showed that MT had no significant effect on *SFRP1*. qPCR results are presented as means ± SEMs of triplicates from three independent experiments, using fold change compared to the control (*n* = 3). a, b, c represent significant differences (*p* < 0.05).

The *β*-*catenin* mRNA level was not significantly different before and after transfection in the control group but was significantly different before and after transfection in the MT group. Before transfection, the *β*-*catenin* mRNA level in the MT group was significantly higher than that in the control group (Figure 10A). There was a significant difference in TCHHL1 mRNA expression between the control group and the other groups, but there was no significant difference in *TCHHL1* mRNA expression between the MT group and the MT-transfected group (Figure 10B). The *SFRP1* mRNA expression level did not change before and after MT treatment but decreased before and after transfection, but the difference was not significant (Figure 10C).

## 4. Discussion

MTNR1a is expressed rhythmically in the hypothalamus, and MTNR1a mRNA expression is higher in the evening than at dawn (44). MTNR1b mRNA is expressed in different tissues and organs of different animals. Migaud et al. (45) detected the expression of MTNR1a in the hypocolliculus papilla precursor and pituitary nodules of sheep, but MTNR1b mRNA was not detected, indicating that MTNR1b does not exist in sheep. At present, most studies on MTNR1b are related to polycystic ovary syndrome (46) and gestational diabetes mellitus (47), and few studies on mammals have been published. The results of Expression of MTNR1a was detected in the hypothalamus and MTNR1b was detected in mouse skin in this study were consistent with the results of Chen et al. (48). In this study, the MTNR1a, MTNR1b and RORα mRNA expression in Inner Mongolia cashmere goat skin tissue in February, April, August, October and December was detected by RT-PCR and *in situ* hybridization. The positive control was the hypothalamus of Inner Mongolia cashmere goat, and MTNR1a mRNA expression was only detected in the hypothalamus tissue. In addition, mouse skin tissue was used as the positive control, and MTNR1b mRNA expression was detected only in mouse skin tissue. MTNR1b mRNA was not expressed in the skin and hypothalamus of Inner Mongolia cashmere goats. However, RORα mRNA expression was detected in the skin tissue at all 5 months.

MT plays a role in immune system regulation, lymphocyte regulation and antitumor activity by binding with RORα, and the expression of RORα mRNA was detected only in the skin tissue of cashmere goats, indicating that skin tissue is the target organ of RORα and RORα is an MT nuclear receptor. However, the expression of RORα in hair follicles is not clear. Therefore, we verified the localization of RORα in skin tissue by *in situ* hybridization. In the control group, RORα had a strong signal in different parts of the hair shaft, hair shaft and hair follicle of cashmere goat skin in February, April, August, October, and December. RORα expression in the implanted group was mainly found in the outer root sheath of the hair shaft and the hair shaft, indicating that RORα may be involved in periodic hair follicle growth. However, whether the change in RORα has a significant effect on the genes related to hair follicle development needs further verification. Therefore, real-time quantitative PCR was used to assess this issue.

August and October are in the long day period, and MT secretion gradually decreases; December, February and April are in the short day period, and MT secretion gradually increases. RORα mRNA expression changes dynamically with periodic changes in MT secretion during the hair follicle development cycle (49). The MT concentration has an antagonistic effect on RORα mRNA expression; that is, when exogenous MT concentration increases, RORα mRNA expression decreases (50), thus regulating the periodic growth of hair follicles. This conclusion is consistent with the results of this study. However, the RORα mRNA expression in February was not consistent with this pattern, which may be caused by RNA degradation. MT implantation promoted RORα expression at the protein level more directly than at the mRNA level. Therefore, further verification of the function of RORα protein in cashmere goat skin hair follicles is needed.

Western blotting and immunofluorescence were used to further verify the effect of MT on RORα expression. Univariate analysis of the experimental results showed that the RORα protein level in the experimental group was higher than that in the control group in February. Because the hair follicles are dormant in February, high levels of RORα protein could be stored for hair follicle redevelopment, indicating that MT implantation may promote RORα protein expression. There was no significant difference in September and December. Immunofluorescence analysis showed that the RORα protein was expressed in February, September and December of the hair follicle development cycle. The RORα protein was expressed in the outer root sheath and dermal papilla of the primary hair follicles and secondary hair follicles in the experimental group but was only expressed in the outer root sheath of the primary and secondary hair follicles in the control group. Therefore, MT could promote villus growth. RORα receptors may bind to the dermal papilla to promote villus growth and development. Hair stems were observed under a fluorescence microscope before and after immunofluorescence, and whether the RORα protein is expressed in hair stems needs further verification.

Zhao Xiaohong (51) and Kang Jian (52) downregulated the expression of RORα in MGC803 cells and fibroblasts by the RNAi technique and found that RORα expression affected the related proteins β-*catenin*, MMP-9, and TIMP-3 and the related gene FGF5. Xi Wenhui (53) transfected MTNR1a into fibroblasts to explore the expression of genes related to hair follicle development and found that the MT membrane receptor was not expressed in skin hair follicles, and only the nuclear receptor RORα was significantly expressed. Therefore, shRNA was linked to an interference vector in this study, and liposome transient transfection technology was used to transfect fibroblasts. The real-time fluorescence quantitative PCR results showed that the expression level of RORα increased with the decrease in MT secretion with exposure to long sunlight, whereas the expression level of RORα decreased with the increase in MT secretion, indicating that MT has a negative regulatory effect on its nuclear receptor RORα, which further verified the research of Chang Zili (27). MT regulates the periodic development of hair follicles by binding its own nuclear receptor RORα. Does RORα expression affect villus development? We screened the *β*-*catenin, SFRP1* and *TCHHL1* genes, which are related to hair follicle development, for verification.

The *β*-*catenin* is a key component of the Wnt/*β*-*catenin* signaling pathway, *SFRP1* mainly acts upstream of Wnt signaling and inhibits *β*-*catenin* phosphorylation (54–56). Liu Wei (57) showed that the expression of *β*-*catenin* was proportional to the hair follicle growth cycle in a study of exogenous MT implantation in cashmere goats. Loss of *β*-*catenin* expression resulted in a block of hair lamella formation and abnormal development of hair follicles, while high expression of *β*-*catenin* promoted the differentiation and development of hair follicle stem cells (58, 59). *TCHHL1* is expressed in the root sheath of hair follicles, which determines the formation of hair follicle morphology and hair shape (60). In this experiment, the *β*-*catenin* mRNA level in the experimental group was significantly higher than that in the control group, and the difference was significant before and after transfection, indicating that RORα expression has an effect on *β*-*catenin* and that MT positively regulates *β*-*catenin* expression through RORα. MT did not affect the expression of *SFRP1*, but the *SFRP1* mRNA level was not significantly decreased after transfection, indicating that the downregulation of RORα expression had almost no effect on the expression of *SFRP1*. The *TCHHL1* mRNA level in the experimental group was significantly lower than that in the control group, and the difference was significant before and after transfection, indicating that downregulation of RORα expression has an impact on *TCHHL1* and that MT regulates *TCHHL1* expression through RORα. RORα regulates the coexpression of *SFRP1*, *β*-*catenin* and *TCHHL1*, We hypothesized that there may be some regulatory relationship between the three genes, which needs to be investigated.

## 5. Conclusion

In this study, the expression patterns and distribution of MT receptors MTNR1a, MTNR1b, and RORα in hair follicles of Inner Mongolia cashmere goats in different periods and different tissues were analyzed. The results showed that MTNRla and MTNRIb were absent in the skin tissues. In the control group, the expression signals of RORα were mainly evident in the hair shaft and outer root sheath of hair shaft, while in the MT group, the expression signals were concentrated in the dermal papilla and outer root sheath of hair shaft. It was confirmed that MT regulates the periodic growth of hair follicles by promoting the expression of nuclear receptor RORα in the dermal papilla, and also found that MT can up-regulates *β*-*catenin* and down-regulates *TCHHL1* related to hair follicle development, and the expression of both genes is down-regulated after inhibiting the expression of RORα. These results provide theoretical basis and data basis for further elucidate the mechanism of MT regulation of secondary hair follicle development.

## Data availability statement

The original contributions presented in the study are included in the article/Supplementary material, further inquiries can be directed to the corresponding author.

## Ethics statement

The animal study was reviewed and approved by the Scientific Research and Academic Ethics Committee of Inner Mongolia Agricultural University and the Biomedical Research Ethics of Inner Mongolia Agricultural University [Approval No. (2020) 056].

## Author contributions

JW (third author) and TZ conceived and designed the study, and obtained, analyzed, and interpreted the data. ZeL drafted the manuscript and revised it. ZeL, JW (second author), QM, T, JL, and ZXW analyzed and interpreted the data. YHZ conceived and designed the study, received funding and oversaw the study. All authors were involved in the design and implementation of the experiment and in the writing of the manuscript.

## Funding

This research was supported by grants from the National Key Research and Development Program of China (2022YED1300200); National Natural Science Foundation of China (32160772 and 31860628); Special Project for Cultivating High Level Achievements in the College of Animal Science (BZX202204); and Innovative Research Team in Universities of Inner Mongolia Autonomous Region (NMGIRT2322).

## Conflict of interest

The authors declare that the research was conducted in the absence of any commercial or financial relationships that could be construed as a potential conflict of interest.

## Publisher’s note

All claims expressed in this article are solely those of the authors and do not necessarily represent those of their affiliated organizations, or those of the publisher, the editors and the reviewers. Any product that may be evaluated in this article, or claim that may be made by its manufacturer, is not guaranteed or endorsed by the publisher.

## Footnotes

^1^https://www.jianguoyun.com/p/DfsDLxQQ287JCxjS64EFIAA
