# Supplementary material for: Melatonin regulates the periodic growth of secondary hair follicles through the nuclear receptor RORα
Source: Front Vet Sci. 2023 Jul 10;10:1203302. doi: 10.3389/fvets.2023.1203302 (PMC10374452; doi:10.3389/fvets.2023.1203302)
Supplement: Supplementary file 1 [file Data_Sheet_1.pdf]

frontiers

Supplementary Material

Melatonin regulates the periodic growth of secondary hair follicles through the nuclear receptor RORαFirst Author*, Co-Author, Co-Author

*** Correspondence:** Yanhong Zhao: 13947196432@163.com

1 Supplementary Figures

A

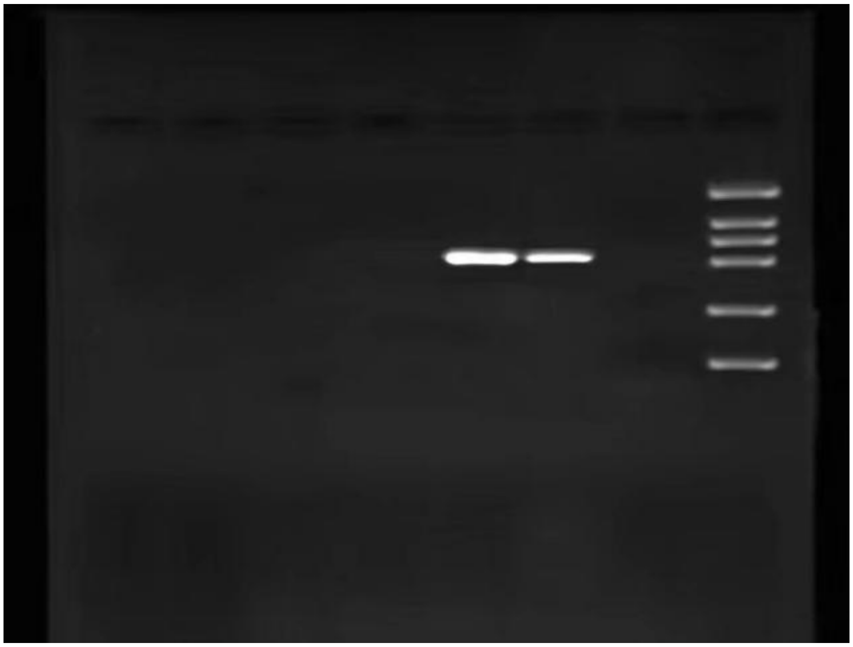

B

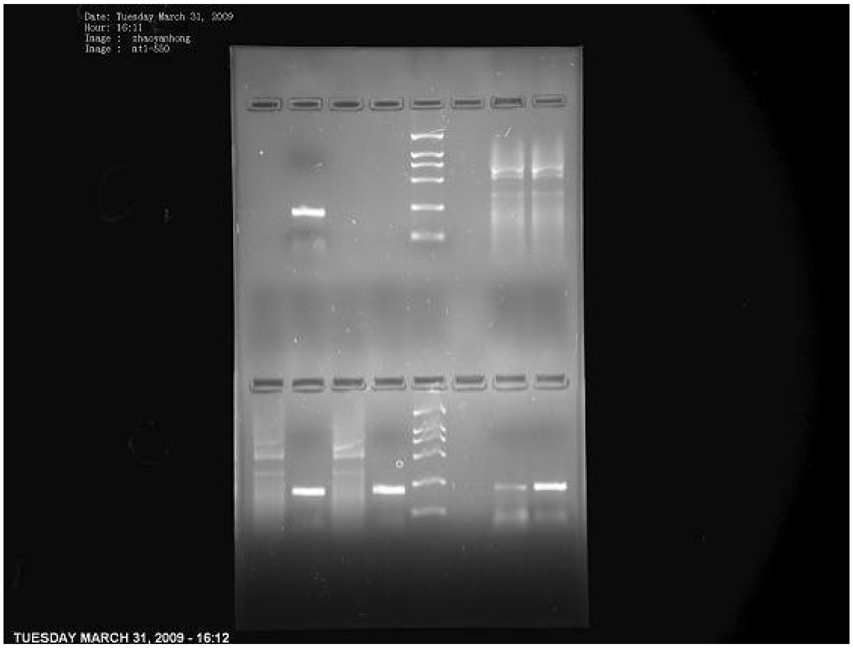
Date: Tuesday March 31, 2009
Hour: 16:11
Image : zhaoyanhong
Image : nt1-550
TUESDAY MARCH 31, 2009 - 16:12

C

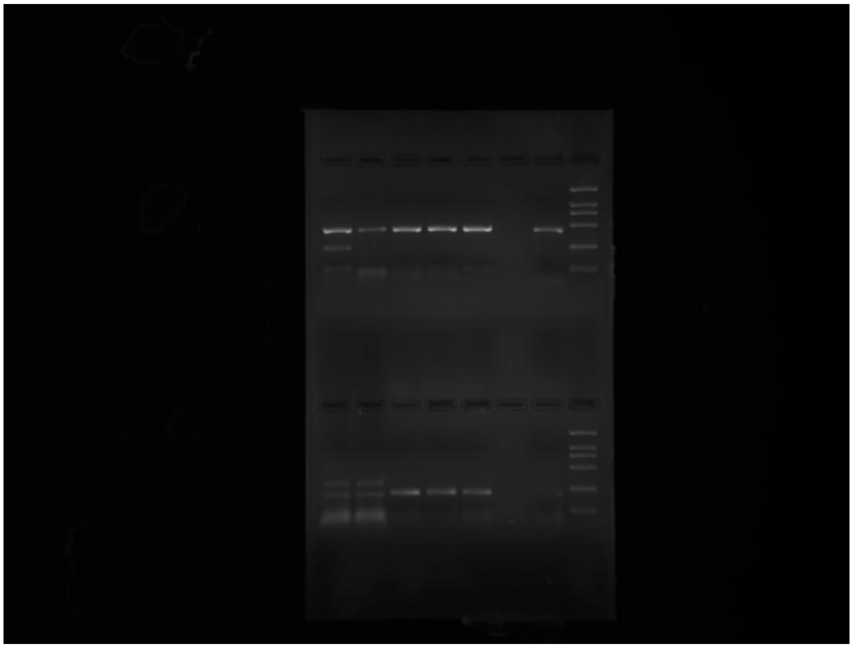

Supplementary Figure 1. Uncropped view of Figure 2 in the article results. (A) Cut the 5 swimlanes on the right side of the original image. (B) Cut the 5 swimlanes on the left side of the original image. (C) Use the entire original image.

A

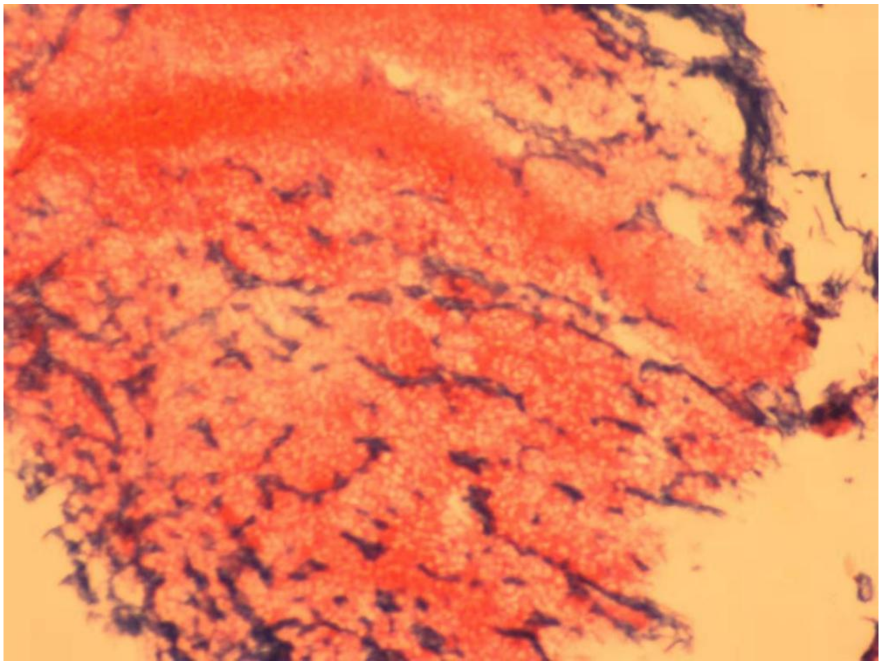

B

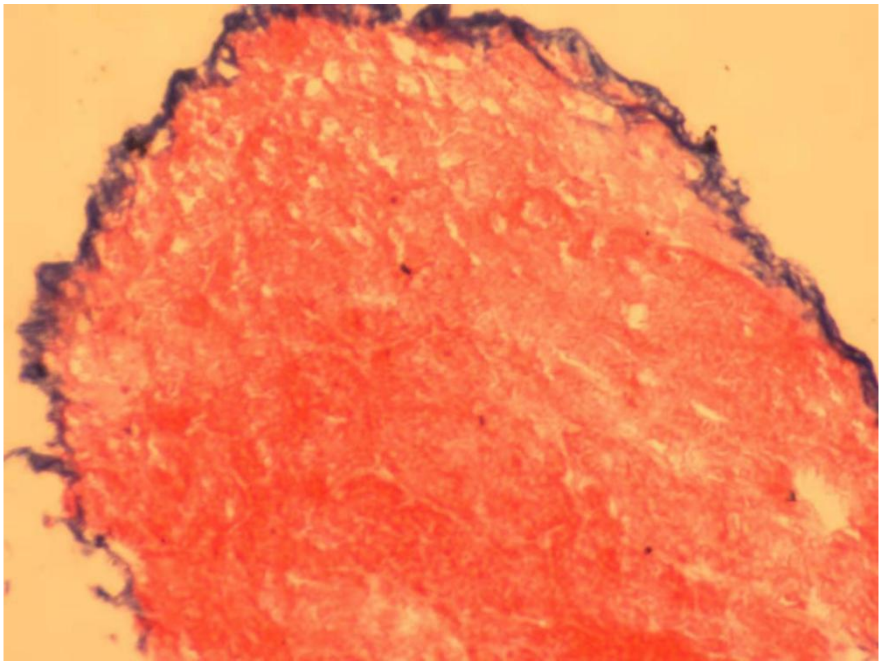

C

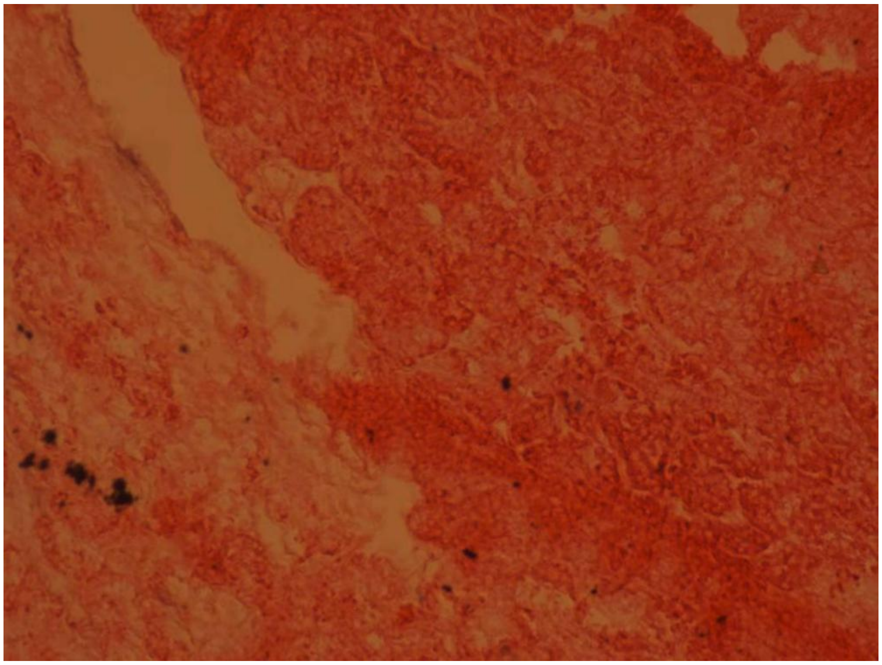

D

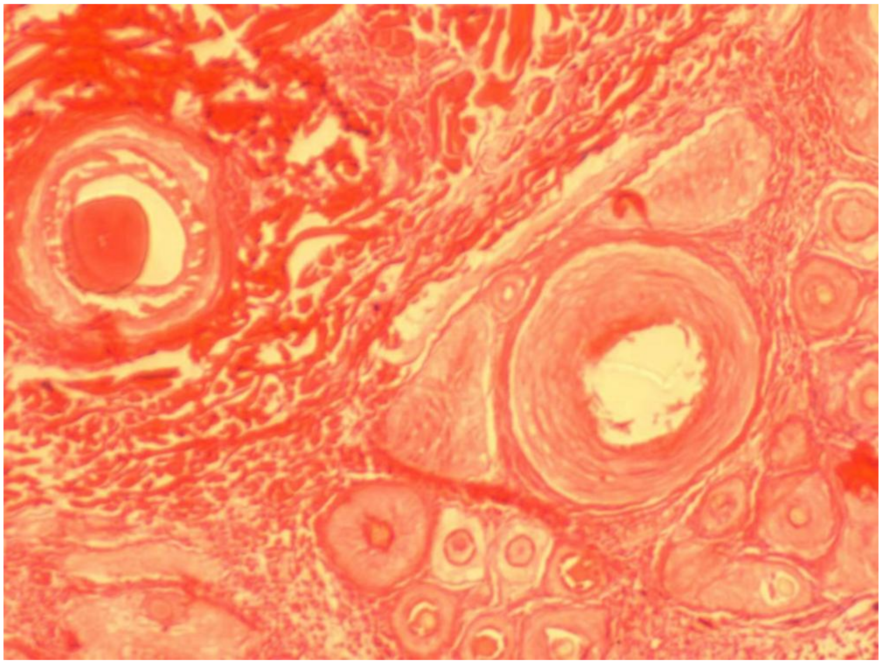

E

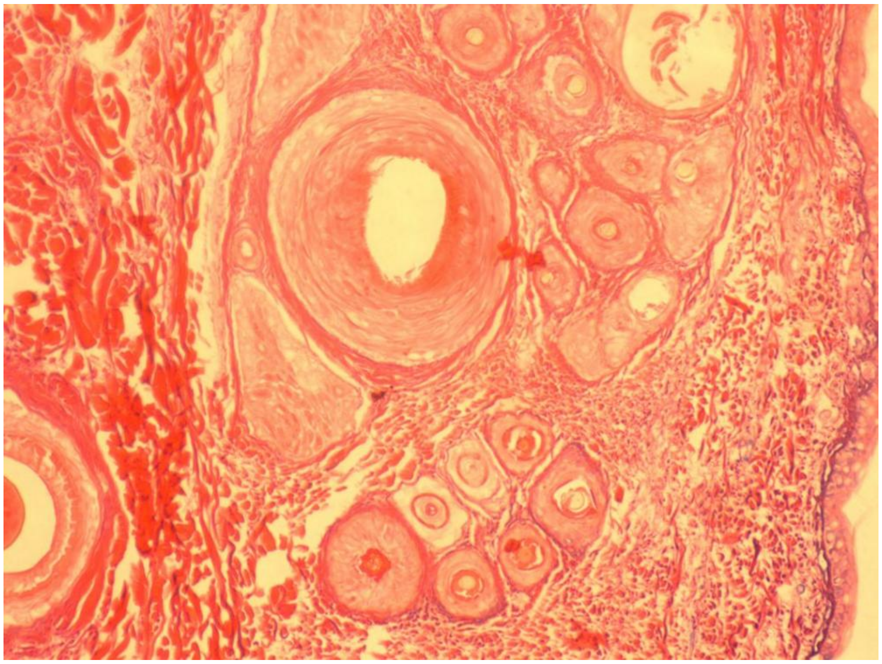

F

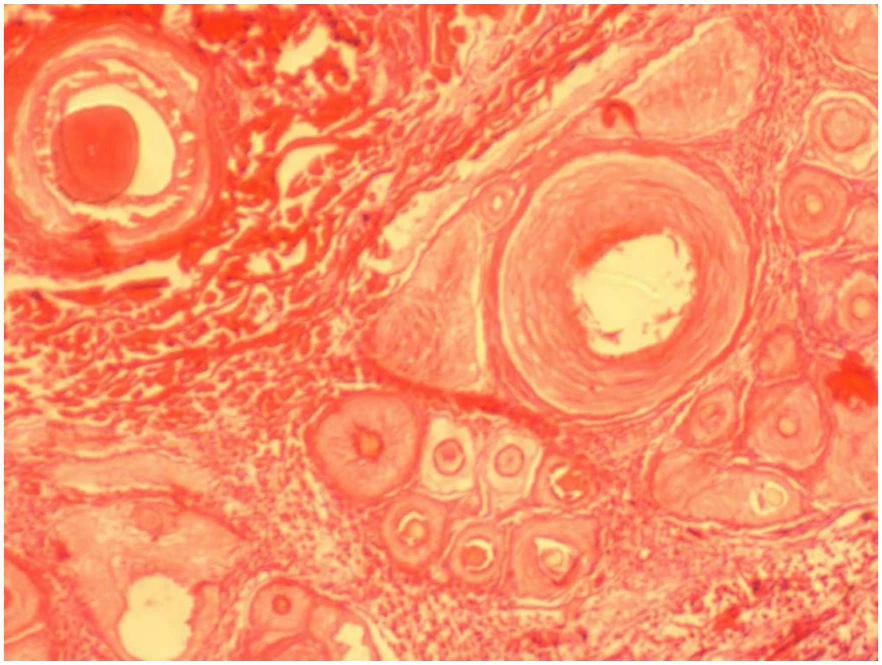

Supplementary Figure 2. Uncropped view of Figure 3 in the article results. (A) Cut the middle and upper part of the original image. (B) Cut the entire center part of the original image. (C) Cut the middle and upper part of the original image. (D) Cut the upper left part of the original image. (E) Cut the middle and upper part of the original image. (F) Cut the upper right part of the original image.

A

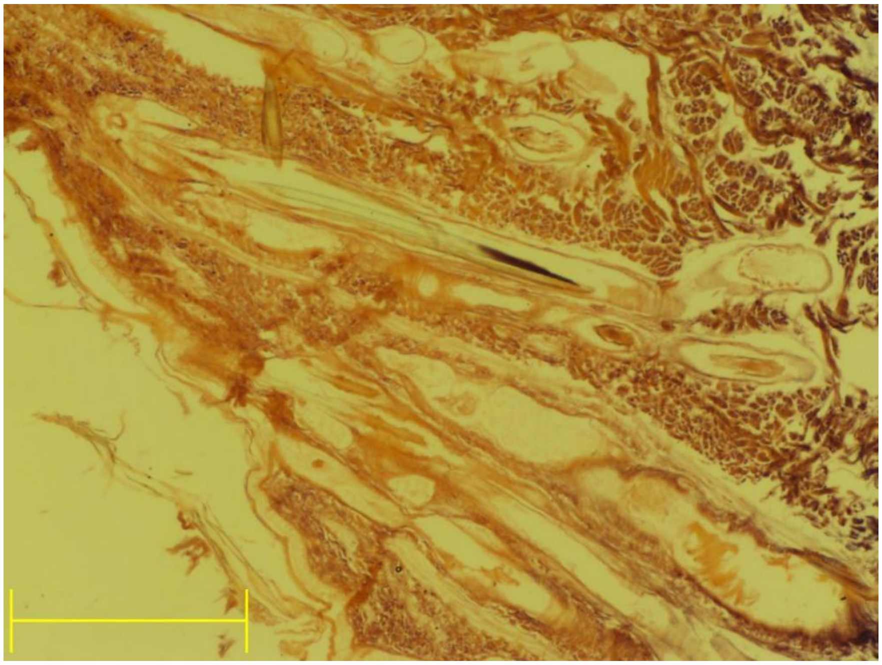

B and C

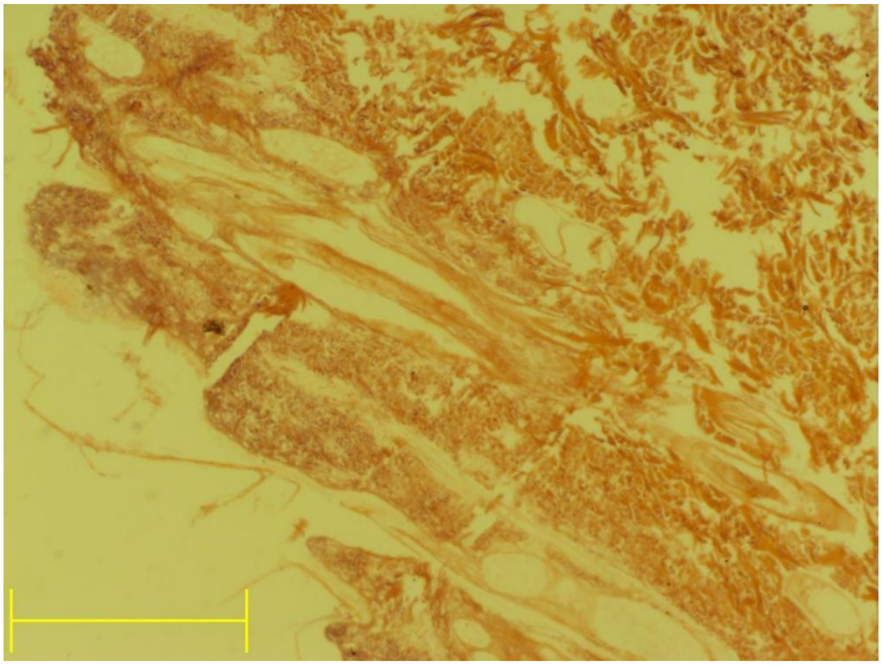

J

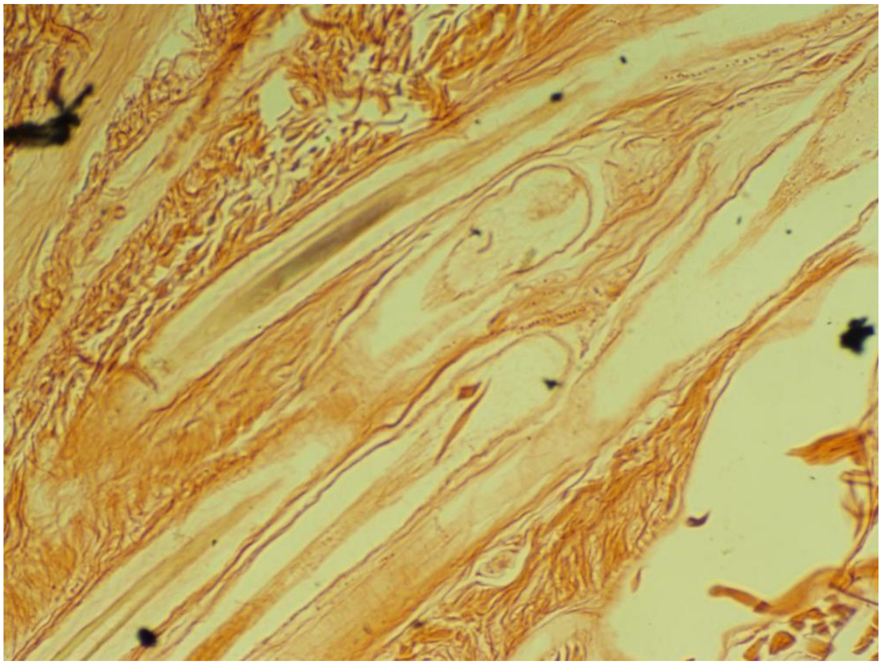

K

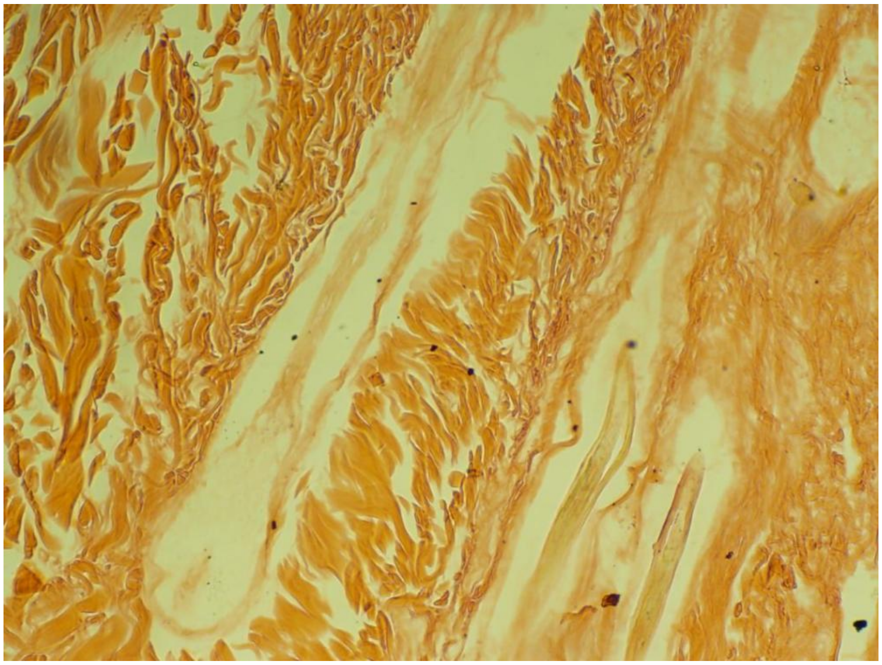

L

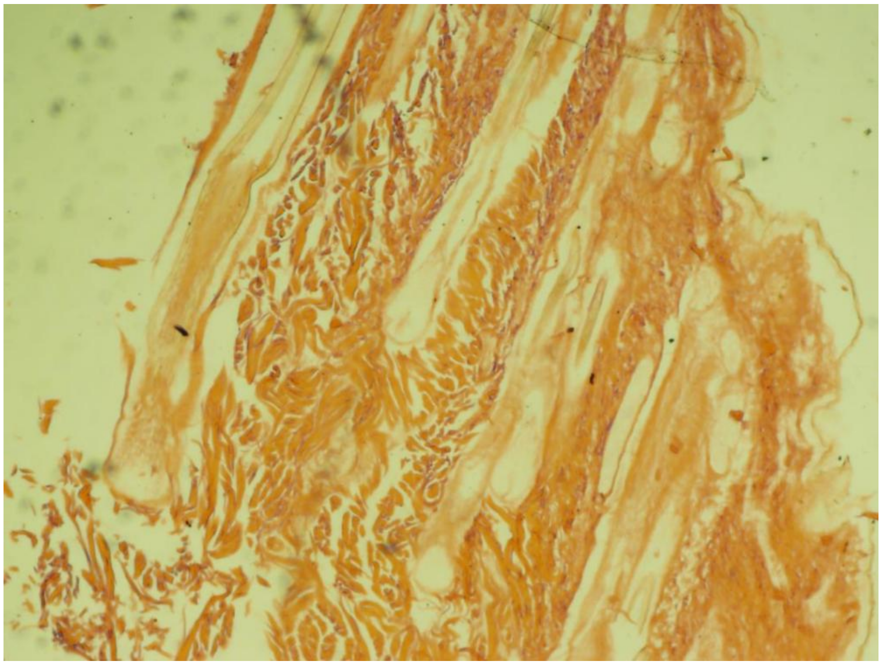

Supplementary Figure 3. Uncropped view of Figure 5 in the article results. (A) Cut the center right part of the original image. (B) Cut the underneath middle part of the original image. (C) Cut the upper left part of the original image. (J) Cut the underneath left part of the original image. (K) Cut the middle left part of the original image. (L) Cut the entire center part of the original image.

A

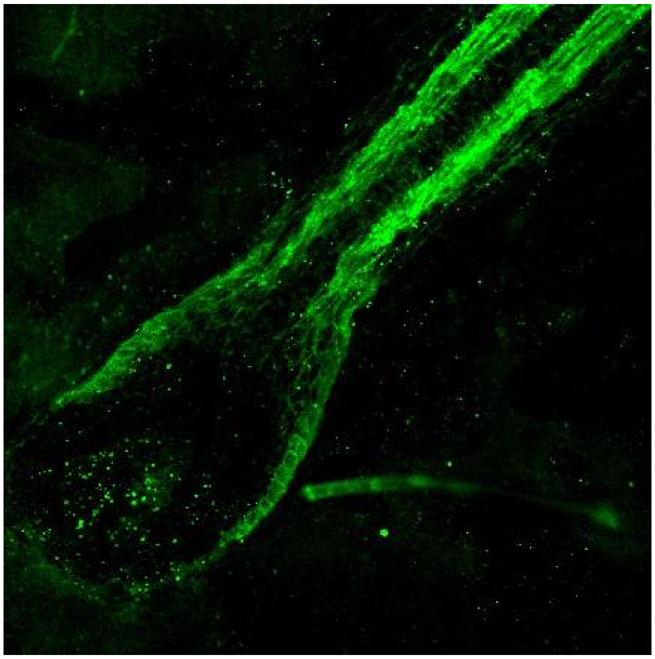

B

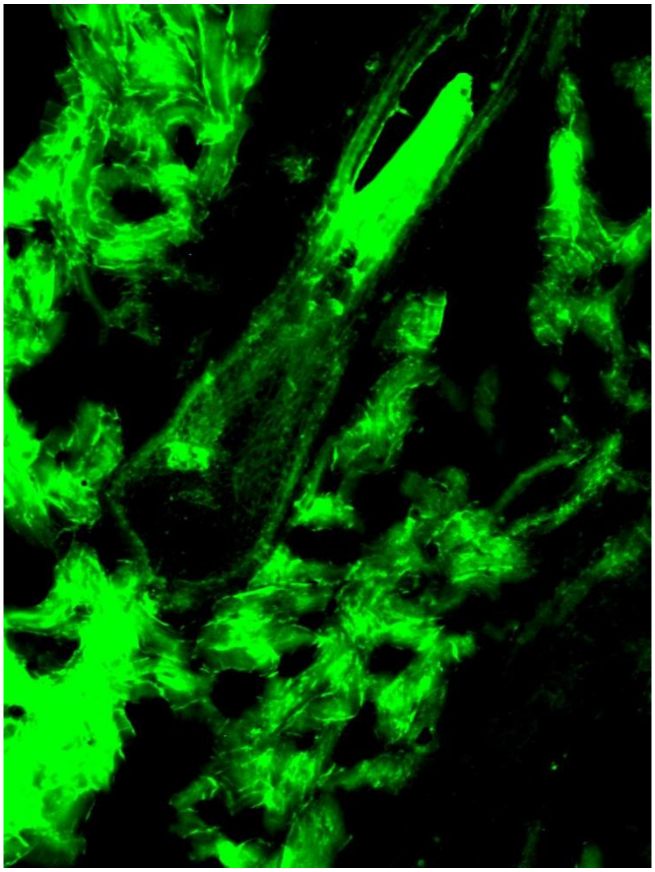

C

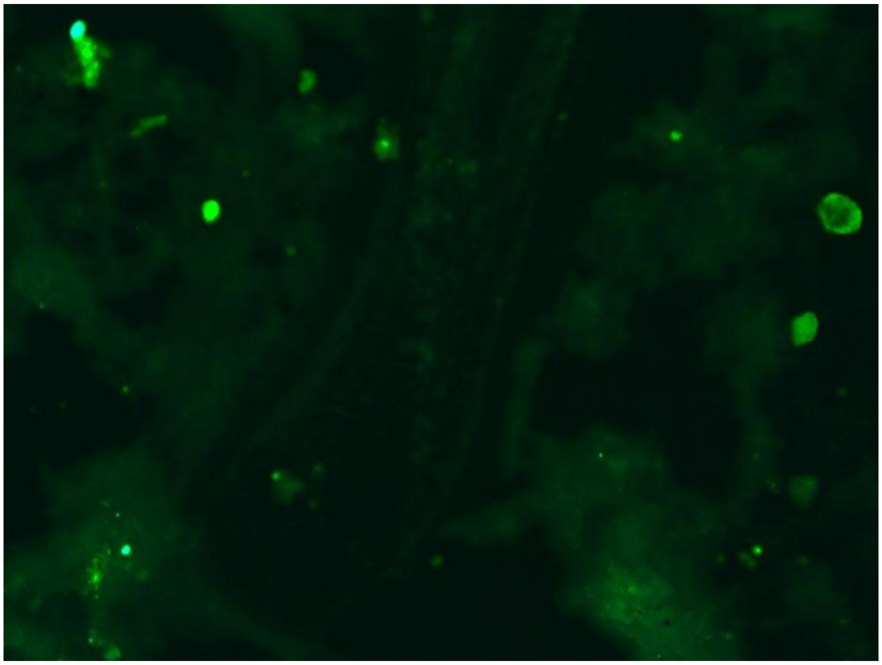

D

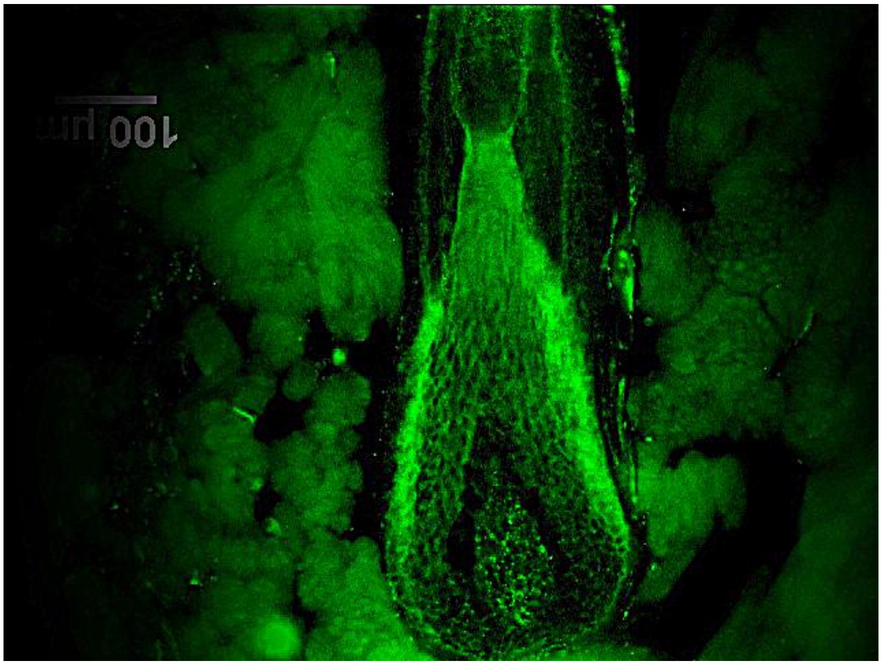

E

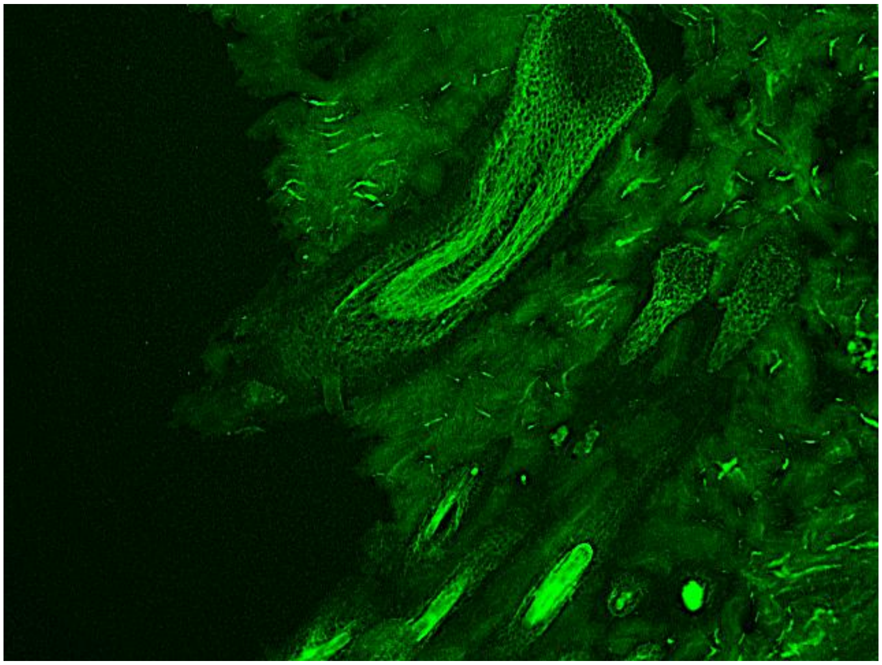

F

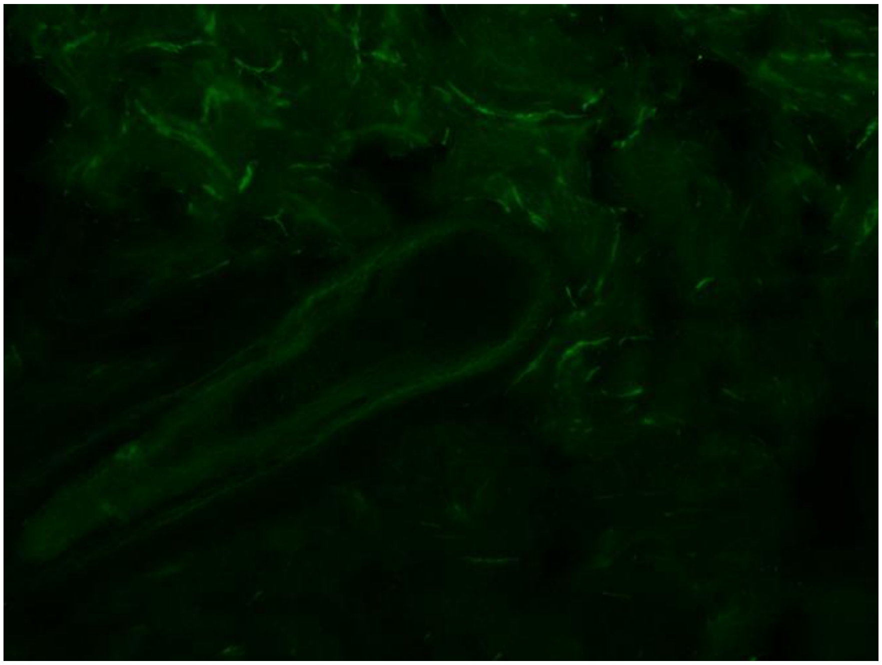

G

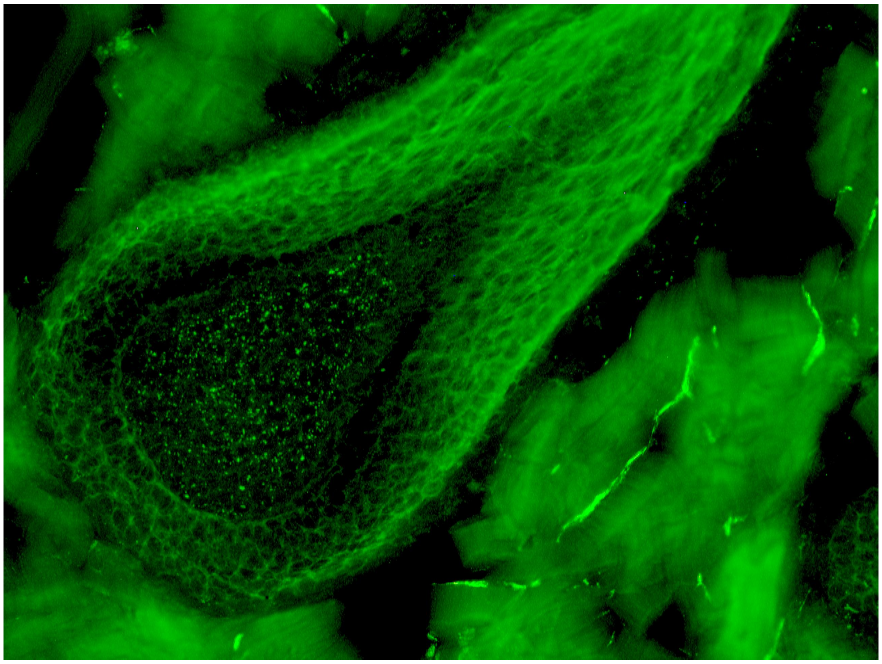

H

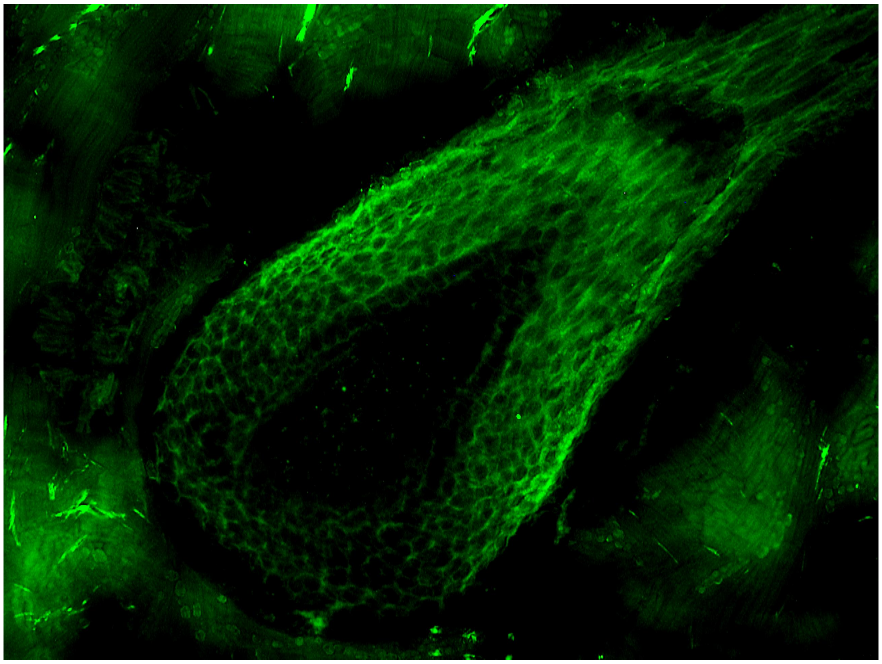

I

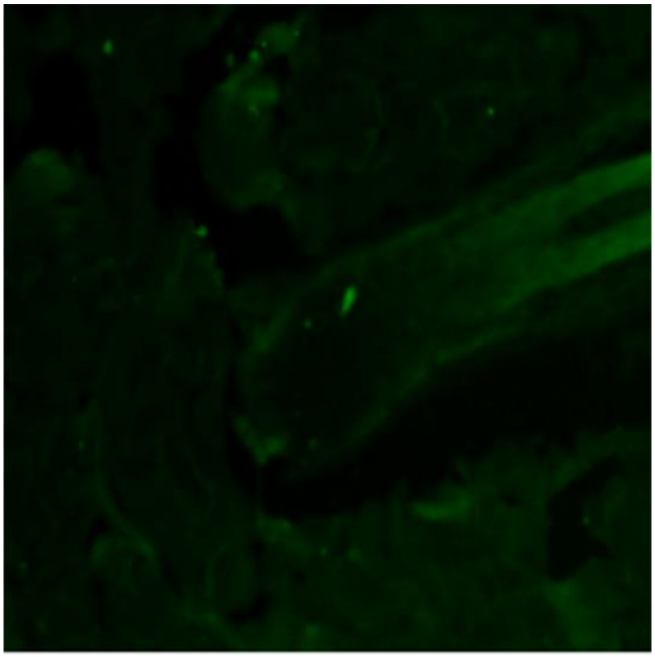

Supplementary Figure 4. Uncropped view of Figure 6 in the article results. (A) Cut the under left part of the original image. (B) Cut the middle part of the original image. (C) Cut the middle part of the original image. (D) Cut the underneath middle part of the original image. (E) Cut the upper middle part of the original image. (F) Cut the underneath left part of the original image. (G) Cut the left part of the original image. (H) Cut the right part of the original image. (I) Use the entire original image.

J

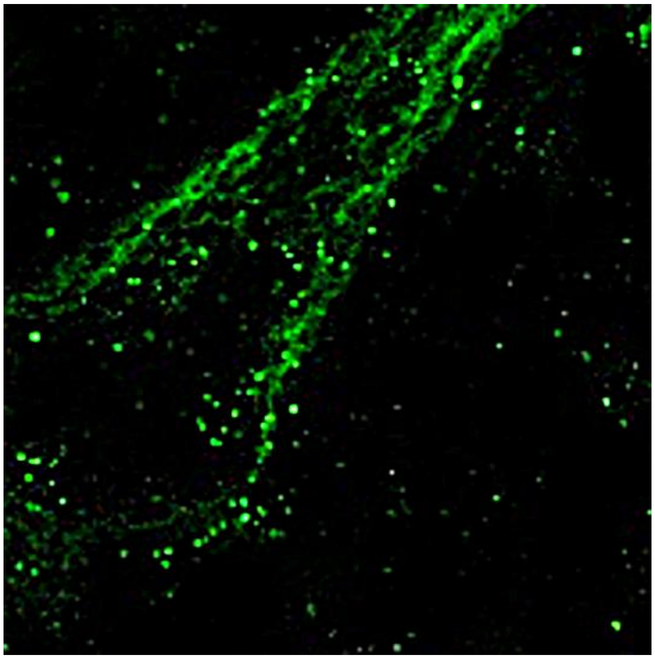

K

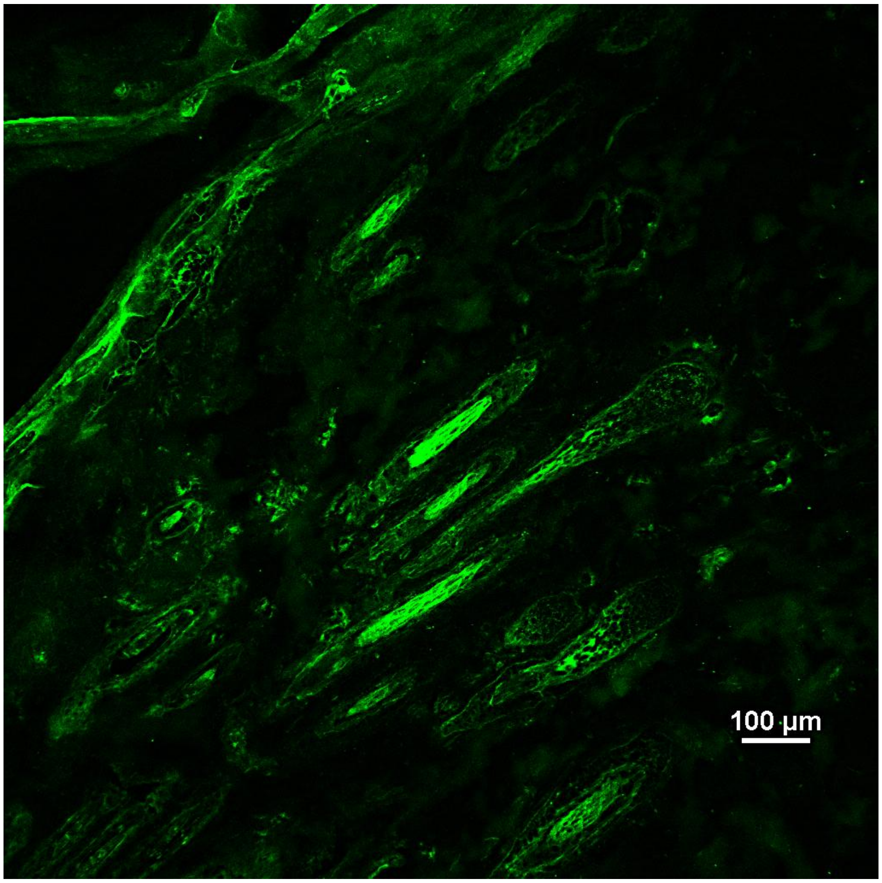

L

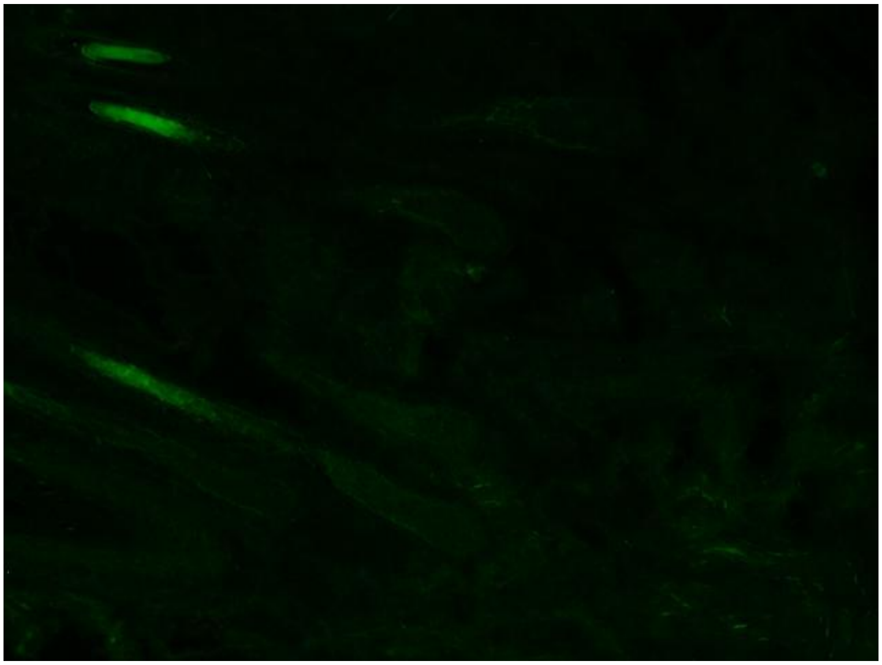

M

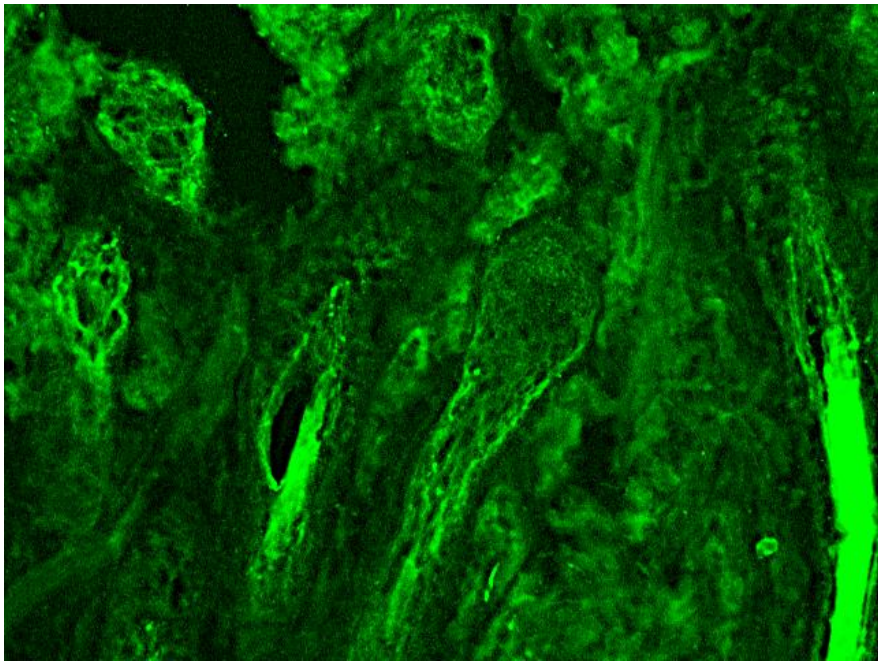

N

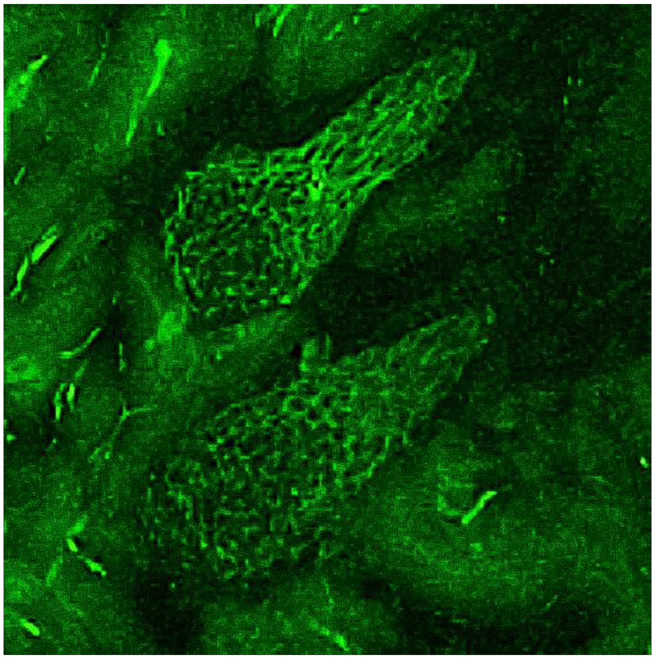

O

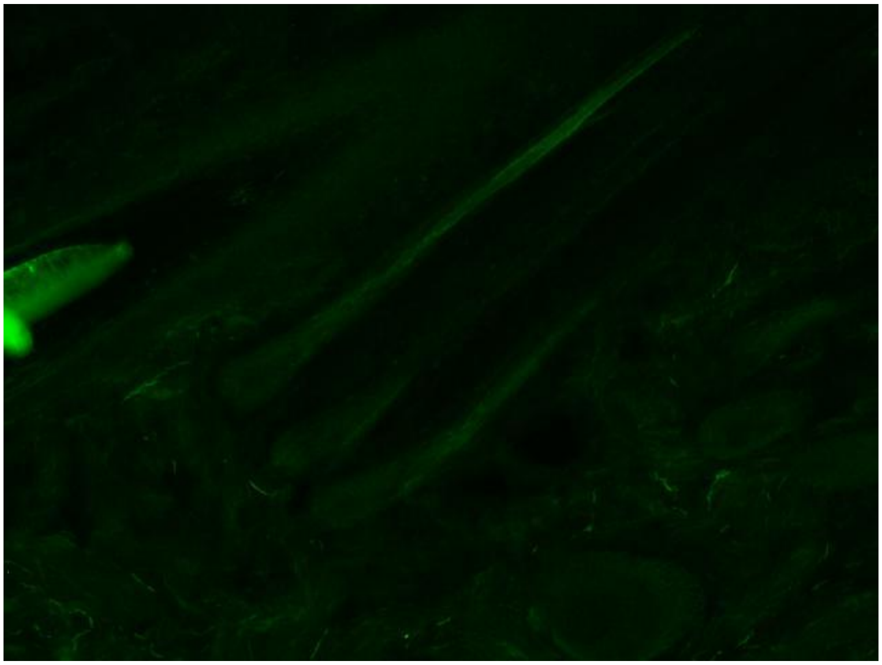

P

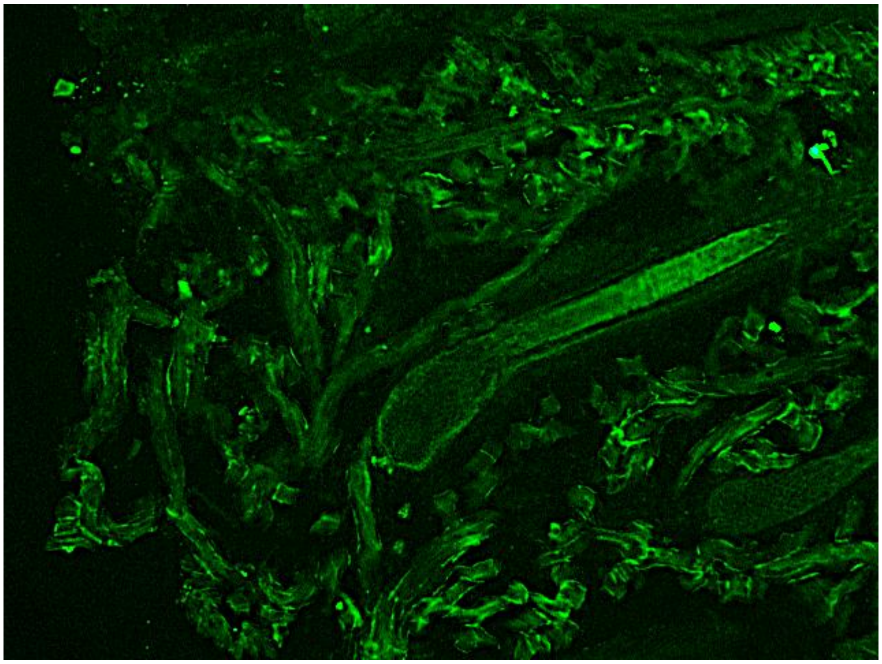

Q

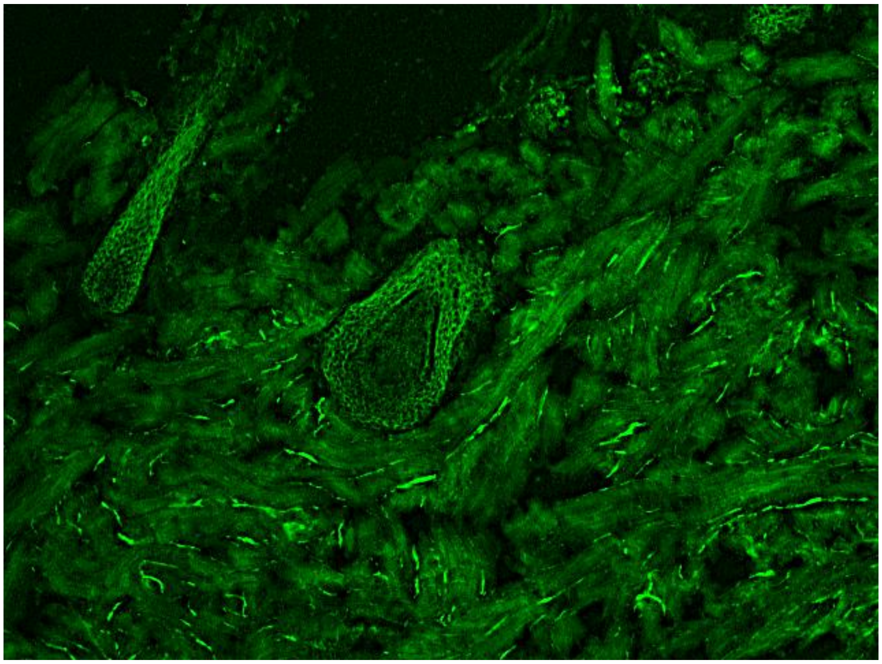

R

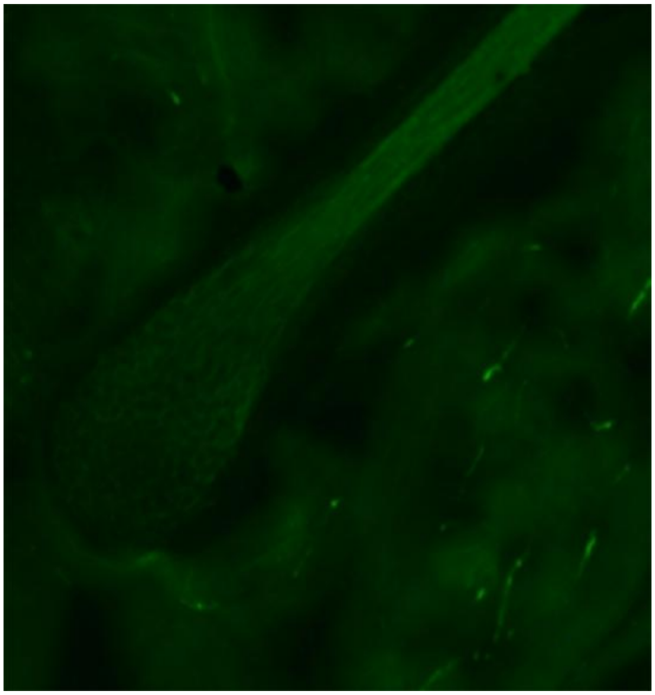

Supplementary Figure 5. Uncropped view of Figure 7 in the article results. (J) Use the entire original image. (K) Cut the middle right part of the original image. (L) Cut the left lower corner of the original image. (M) Cut the middle right part of the original image. (N) Use the entire original image. (O) Cut the center part of the original image. (P) Cut the center part of the original image. (Q) Cut the left upper corner part of the original image. (R) Use the entire original image.

A

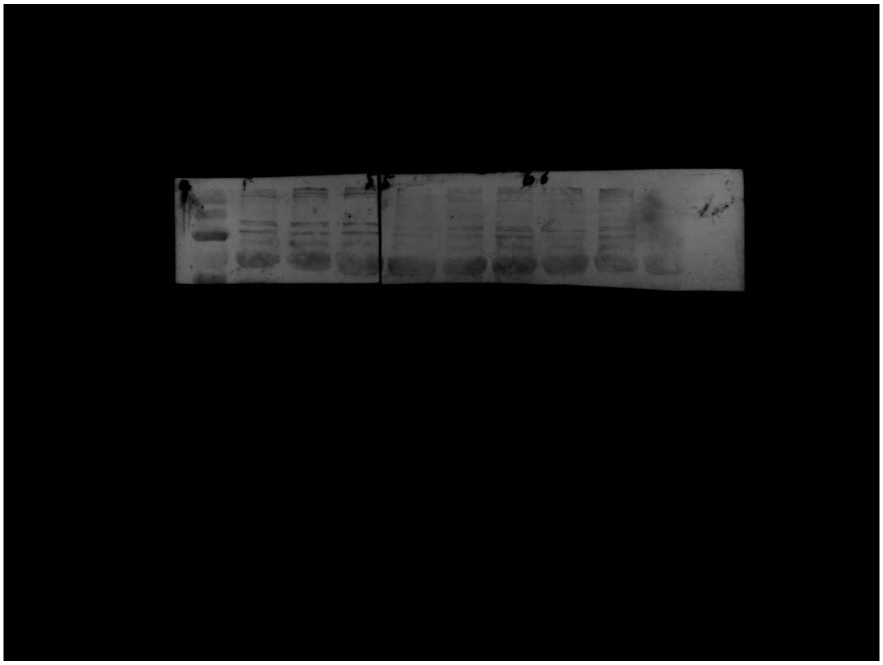

B

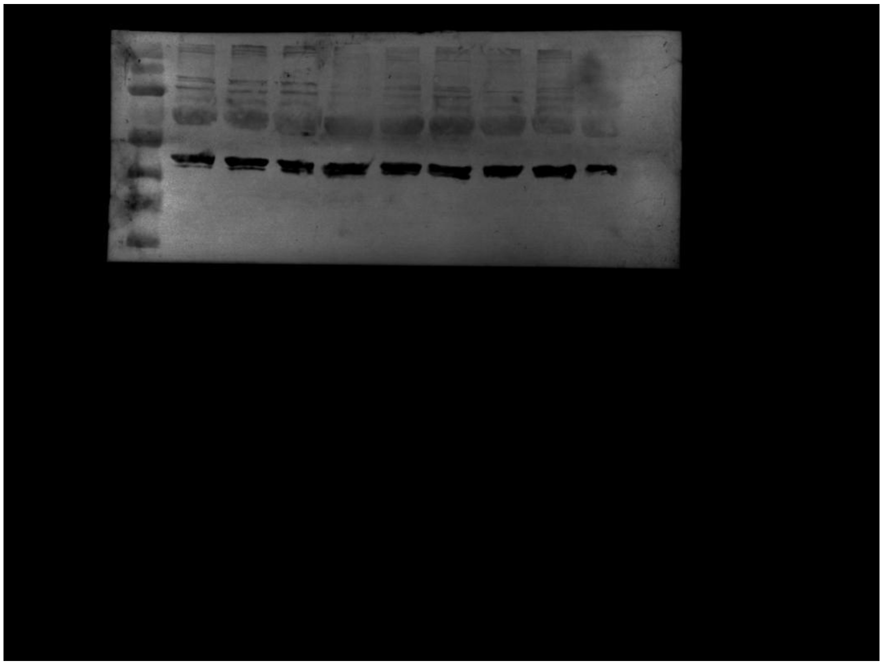

Supplementary Figure 6. Uncropped view of Figure 8 in the article results. (A) Cut the 6 swimlanes on the right side of the original image. (B) Cut the 6 lanes on the right side of the original image.
